# T-follicular regulatory cells expand to control germinal center plasma cell output but fail to curb autoreactivity

**DOI:** 10.1016/j.isci.2024.110887

**Published:** 2024-09-04

**Authors:** Cecilia Fahlquist-Hagert, Thomas Rea Wittenborn, Mattias Krogh Pedersen, Lisbeth Jensen, Søren Egedal Degn

**Affiliations:** 1 Laboratory for Lymphocyte Biology, Department of Biomedicine, Aarhus University, 8000 Aarhus C, Denmark

**Keywords:** Immunology, Components of the immune system, Cell biology

## Abstract

Autoantibodies generated in germinal centers (GCs) contribute to the pathogenesis of autoimmune diseases. GCs are controlled by specialized FoxP3+ T-follicular regulatory cells (Tfr), but their role in established autoimmunity is unclear. We generated autoimmune bone marrow chimeras in which Tfr could be specifically ablated by diphtheria toxin. Furthermore, we tracked the clonal persistence and evolution of Tfr populations using Confetti reporters. Ablation of Tfr caused increased early plasma cell output, but longer-term ablation did not increase plasma cell levels beyond those of Tfr-sufficient controls, suggesting that Tfr fail to contain chronic autoreactive GC responses. In agreement, Tfr were robustly induced in early autoreactive GCs but then waned. Moreover, we observed polyclonal Tfr expansion when ablating part of the Tfr subset. Hence, under homeostatic conditions, a polyclonal population of Tfr operates to control autoreactivity by limiting the output of plasma cells from GCs, but in chronic autoimmunity, this mechanism fails.

## Highlights

• Autoimmune GCs harbor a polyclonal Tfr population
• Short-term Tfr ablation causes increased output of plasma cells from autoimmune GCs
• Long-term Tfr ablation does not exacerbate plasma cell output from autoimmune GCs
• Partial Tfr ablation during an autoimmune response causes polyclonal Tfr expansion

## Graphical abstract

**Figure:**
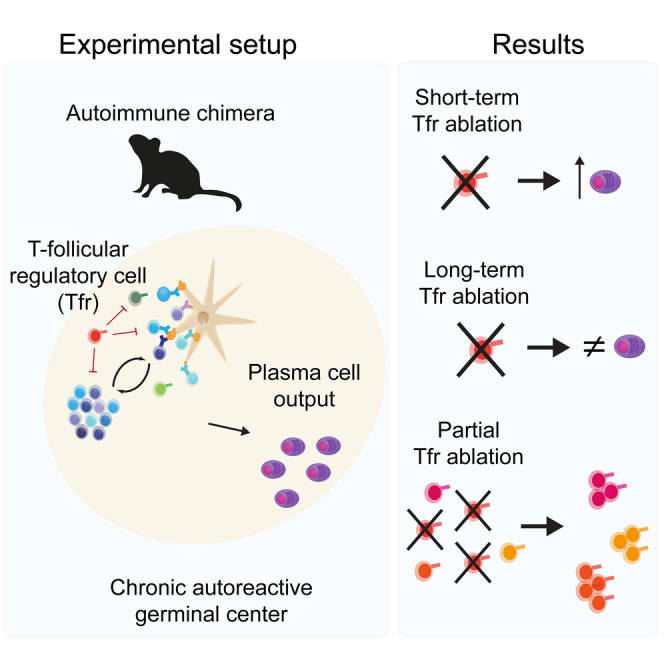

## Introduction

Germinal centers (GCs) are the sites of affinity maturation of the antibody response, a process whereby B cells undergo clonal expansion, somatic hypermutation of their immunoglobulin genes, and selection based on antigen affinity.^1^ These “induced microanatomical structures” form in secondary lymphoid organs in response to antigens, which may be derived from exogenous, e.g., infectious, sources, or from the host in the setting of autoimmunity. The GCs are seeded by B-cell clones that have encountered their cognate antigen and have received help from primed CD4 T-helper cells at the T-B border.^2^ B and T cells co-migrate into the follicle, where the B cells proliferate vigorously to form a dark zone of centroblasts, whereas the T cells take up residence in the light zone and are now termed T-follicular helper cells (Tfh). The centroblasts subsequently undergo activation-induced cytidine deaminase-directed mutagenesis, which introduces subtle variations in their paratopes.^3^ Mutated daughter cells migrate into the light zone, where they probe antigens retained by follicular dendritic cells, and are now termed centrocytes.^4^ The centrocytes that have stochastically obtained higher-affinity B-cell receptors are able to competitively take up antigens, and present antigen-derived peptides on their MHC class II (MHCII) molecules, in turn allowing their selection by Tfh.^5^^,^^6^^,^^7^ There is an absolute requirement for peptide-MHCII presentation for entry of B cells into the GC; however, the selection of centrocytes appears to rest on a combination of B-cell receptor signaling and subsequent peptide-MHCII selection, both in foreign antigen-directed responses^8^ and autoimmunity.^9^

The requirement for peptide-MHCII selection at every cycle ensures that the GC B cells retain their focus on the eliciting antigen throughout their affinity maturation process. Nonetheless, the possibility remains that T-cell tolerance can be broken, allowing the emergence of Tfh that would support autoreactive GC B-cell responses. A third GC lymphocyte subset, the T-follicular regulatory cell (Tfr), seems to counter this challenge.^10^ Discovered in 2011, this FoxP3-expressing T-cell subset emerging from natural Treg precursors was found to be able to regulate the GC reaction, suppressing the magnitude of the GC response and limiting antibody production to T-dependent antigens.^11^^,^^12^^,^^13^ Tfr have since been found to restrict and focus the breadth of the antibody response, in some cases supporting the production of specific antibodies while limiting inadvertent responses.^14^ Like conventional Tregs, Tfr are characterized by the expression of FoxP3, but they are distinguished from these by the expression of the follicular chemokine receptor CXCR5 and the GC transcription factor Bcl-6.^11^^,^^12^^,^^13^ For this reason, a transgenic strategy employing FoxP3-Cre combined with Bcl6^flx/flx^ has been widely leveraged to specifically block Tfr development, and this was reported to promote autoimmunity.^15^ However, it was recently reported that Tfh also upregulates FoxP3 in end-stage GCs to shut off the GC response.^16^ This has cast doubt upon the prior findings relying on this transgenic strategy, because in the FoxP3-Cre Bcl6^flx/flx^ model, Tfh would be prevented from shutting down GCs, providing an alternative explanation for the earlier reported findings. It remains an open question to what extent Tfr populations observed in earlier studies included Tfh that had upregulated FoxP3. Nonetheless, the observation that by and large, the repertoire of Tfr is distinct from that of Tfh^17^ indicates a distinct, bona fide subset of Tfr.

The peripheral Treg compartment is known to be homeostatically controlled, and partial ablation has demonstrated that it relatively rapidly (∼2 weeks) recovers in a thymus-independent fashion by the homeostatic proliferation of surviving, non-depleted Tregs.^18^ The dynamic regulation of Tfrs is less well studied, particularly in the context of autoimmunity. Here, we asked to what extent Tfr are involved in controlling chronic autoimmunity, and whether Tfr display clonal selection and expansion. We find that loss of Tfr cells leads to increased plasma cell output from autoreactive GCs at early, but not late timepoints, indicating the inability of these cells to control an ongoing autoimmune response. Using the Confetti system for clonal lineage tracing, we find that Tfr cells maintain clonal diversity over time and can accommodate partial deletion through polyclonal expansion.

## Results

### Autoimmune model allowing temporally controlled, specific ablation of T-follicular regulatory cells

We previously established a mixed bone marrow chimera model of B-cell driven autoreactivity, whereby one bone marrow compartment carrying an autoreactive knock-in BCR (564Igi) drives a break-of-tolerance, which elicits epitope spreading in wild-type B cells derived from a normal bone marrow compartment.^19^ The knock-in BCR cells carry a dual homozygous knock-in of the heavy and kappa light chains (H^ki/ki^ K^ki/ki^), precluding escape of autoreactivity by receptor editing.^20^ Control chimeras carrying heavy chain knock-in only (H^ki/ki^ K^wt/wt^) do not display spontaneous GCs, elevated plasmablast/cell frequencies, nor autoantibodies, demonstrating that it is the presence of cells carrying the autoreactive BCR that drives the autoimmune phenotype.^19^^,^^21^ We have shown that the initial break-of-tolerance in the T-cell compartment occurs outside GCs, requires antigen-presentation by the eliciting B-cell clone, and that epitope spreading subsequently progresses through GCs.^21^ Both Tfh and Tfr have been shown to clonally expand, and display repertoires that are distinct from those of non-autoimmune chimeras immunized with foreign antigen,^22^ suggesting that Tfr are expanding to control the autoreactive GC populations in this model.

**Figure 1:**
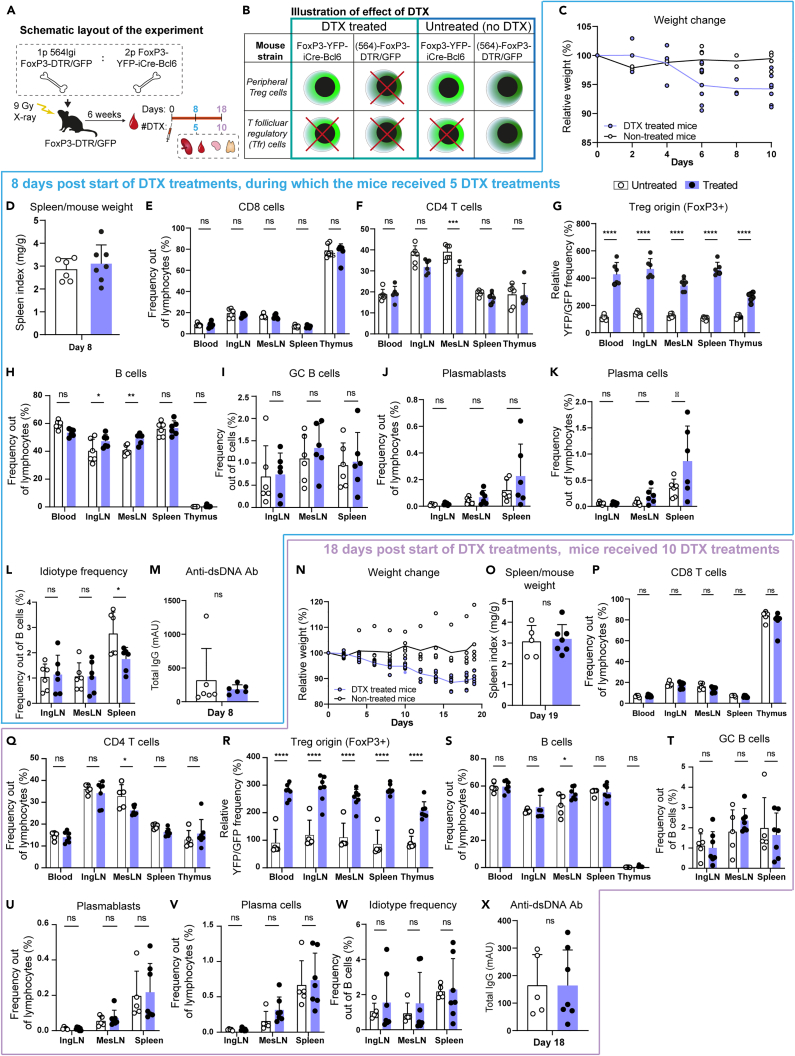
Depletion of T-follicular regulatory cells (Tfr) does not alter the frequency of germinal center (GC) B cells (A) Schematic representation of the experimental layout. Chimeras were rested for 6 weeks, then bled to verify chimerism (Day 0), and DTX was injected every 3 days (or not injected, untreated). Cohorts were analyzed on day 8 (5 DTX injections) or day 18 (10 DTX injections). (B) Schematic representation of the resulting ablation of the regulatory T cells upon DTX treatment. The effect of Tfr ablation was measured after 8 (C–M) or 18 days (N–X). (C and N) Relative weight change of the mice compared to the start of the experiment for untreated (*n* = 6 and 5, respectively) and DTX treated (*n* = 6 and 7, respectively). Circles indicate the weight of individual mice and curves represent the mean. (D and O) Spleen weight relative to body weight. (E and P) Frequency of CD8 T cells in the blood, inguinal and mesenteric lymph nodes (IngLN and MesLN, respectively), spleen, and thymus. (F and Q) Frequency of CD4 T cells. (G and R) Relative ratio of YFP (Tfr block) over GFP (DTX-ablatable) cells, reflecting the reconstitution with 564Igi-FoxP3-DTR/GFP versus FoxP3-iCre/YFP Bcl6^flx/flx^ donor marrow and stability hereof in uninjected controls and the efficacy of the ablation upon DTX treatment. (H and S) Frequency of B cells. (I and T) Frequency of GC B cells. (J and U) Frequency of plasmablasts. (K and V) Frequency of plasma cells. (L and W) Frequency of idiotype (9D11) positive B cells. (M and X) Levels of anti-dsDNA Ab. In all bar graphs, circles show values for individual mice, and bars indicate mean ± SD for n = 5–7 mice per group, pooled from 2 independent experiments for DTX5 and one experiment for DTX10. Statistical significance was given for two-way ANOVA with Šidák’s post-test for (E–L) and (P–W), and Wilcoxon matched-pairs signed rank test for (D, M, O, and X). Significance indicated as ns (not significant) *p* > 0.05; ∗*p* < 0.05; ∗∗*p* < 0.01; ∗∗∗*p* < 0.001; ∗∗∗∗*p* < 0.0001. In all instances, “days” refer to days from the first DTX injection. See also Figures S1–S4.

To test this hypothesis, we sought to establish mixed chimeras, in which we could perform temporally controlled ablation of the Tfr population. Accordingly, we crossed the 564Igi BCR knock-in line to a FoxP3-GFP/DTR line, in which all Tregs, including Tfr, express green fluorescent protein (GFP) fused to the human diphtheria toxin receptor (DTR).^23^ We furthermore crossed a FoxP3-YFP-Cre line, in which again all Tregs, including Tfr, express a yellow fluorescent protein (YFP) fused to a codon optimized Cre recombinase (iCre),^24^ with a Bcl6^flx/flx^ line, in which exons 7–9 of the Bcl6 gene are flanked by *loxP* sites.^25^ In mixed chimeras reconstituted with the 564Igi FoxP3-GFP/DTR driver combined with the FoxP3-YFP-Cre Bcl6^flx/flx^ line (Figure 1A), all Tregs, including Tfr, from the former would be ablatable by diphtheria toxin administration, whereas the latter would only be able to contribute peripheral Tregs, but not Tfr (Figure 1B). This would allow controlled, temporal, and specific ablation of Tfr.

### Establishment of optimal diphtheria toxin dose for the ablation of T-follicular regulatory cells

Although the sensitivity of murine cells to DTX is several orders of magnitude lower than that of cells expressing the human DTR, DTX has been reported to exert unspecific toxicity in mice, which may vary from batch to batch.^26^ Therefore, we first titrated our DTX in a cohort of non-susceptible WT C57BL/6J mice and a cohort of susceptible FoxP3-GFP/DTR mice (Figure S1). This revealed non-specific toxicity at doses of 1 and 0.5 μg, whereas wild-type mice were unaffected at 0.25 μg (for mice with starting weights of ∼25–30 g, corresponding to around 33–40, 17–20, and 8–10 μg/kg, respectively). Accordingly, this latter dose was chosen for subsequent experiments.

### Mildly relaxed control of plasma cell output upon the short-term ablation of T-follicular regulatory cells

Chimeras were established as outlined in the experimental overview in Figure 1A, and blood was drawn at 6 weeks post reconstitution, and the chimerism was determined (Figure S2). Having verified adequate chimerism and normal cell frequencies across compartments, DTX treatment was initiated in half of the chimeras, whereas the other half was left untreated. For treatment, mice were given 0.25 μg DTX i.p. 3 times a week, and their weight compared to untreated controls was monitored. As can be seen, DTX treated mice lost about 5% of body weight by day 8 (Figure 1C), and around 10% of body weight by day 18 of treatment (Figure 1N). Spleen weight relative to total body weight remained constant for the duration of the experiment and was comparable between the two groups (Figures 1D and 1O). The frequency of CD8 T cells was comparable between groups at both time points (Figures 1E and 1P, full gating strategy in Figure S3A), whereas the frequency of CD4 T cells was slightly reduced in mesenteric lymph nodes (MesLN) of the treated group (Figures 1F and 1Q). Looking at the ratio of YFP/GFP as a readout of the origin of Tregs, it was slightly above 1:1 (100%) in the untreated group, as expected from the 2:1 input of YFP to GFP bone marrow into GFP recipients (Figures 1G and 1R, gating examples in Figure S4). However, in the DTX treated group, this was skewed to >4:1 (400%) and ∼3:1 (300%) at the 8 and 18-day time-point, respectively, across blood, inguinal lymph nodes (IngLN), MesLN, and spleen, demonstrating the efficacy of the DTX-mediated ablation of the GFP+ cells. Interestingly, the ratio was lower in the thymus, likely due to a continuous output of T cells during the treatment. Nonetheless, we concluded, that the ablation of GFP+ cells was effective, and there was a potential compensatory homeostatic expansion of YFP+ cells, which could serve regular Treg functions, but were blocked from becoming Tfr. We saw a slight increase in B cells in the treated group (Figures 1H and 1S), which could be explained as a reciprocal effect of the slight decrease in the CD4 T-cell compartment. More importantly, GC B-cell frequencies were comparable between the two groups at both time points (Figures 1I and 1T), and plasmablast levels were indistinguishable (Figures 1J and 1U), whereas there was a small but significant increase in plasma cell levels in the spleen at the early (Figure 1K), but not late (Figure 1V) time point. This held true also if plasmablast and plasma cell levels were normalized to B-cell frequencies (Figures S3B and S3C). Of note, we relied on CD138 and B220 to gate plasmablasts (CD138+B220+) and plasma cells (CD138+B220-), respectively, out of live, singlet lymphocytes. Although CD138 (Syndecan-1) is a canonical plasma marker, it has recently been reported to also be upregulated on a subset of atypical T cells.^27^^,^^28^ As most of these atypical T cells are also CD4 and CD8 double-negative, we could not directly exclude these in our gating strategy, however, analysis of forward and side scatter indicated that the bulk of the gated cells were significantly larger and more granular than T lymphocytes (Figure S3D). The frequency of cells carrying the idiotypic receptor (9D11) was also slightly elevated in the spleen at the early time point (Figure 1L), but not the late (Figure 1W). Taken together, this indicated a mildly relaxed control of plasma cell output and autoreactive B-cell selection in the spleen upon the timed ablation of Tfr. Yet this did not result in a measurable increased output of anti-dsDNA autoantibodies at the late time point (Figure 1X).

### No effect of long-term T-follicular regulatory cell ablation in chronic autoimmunity

**Figure 2:**
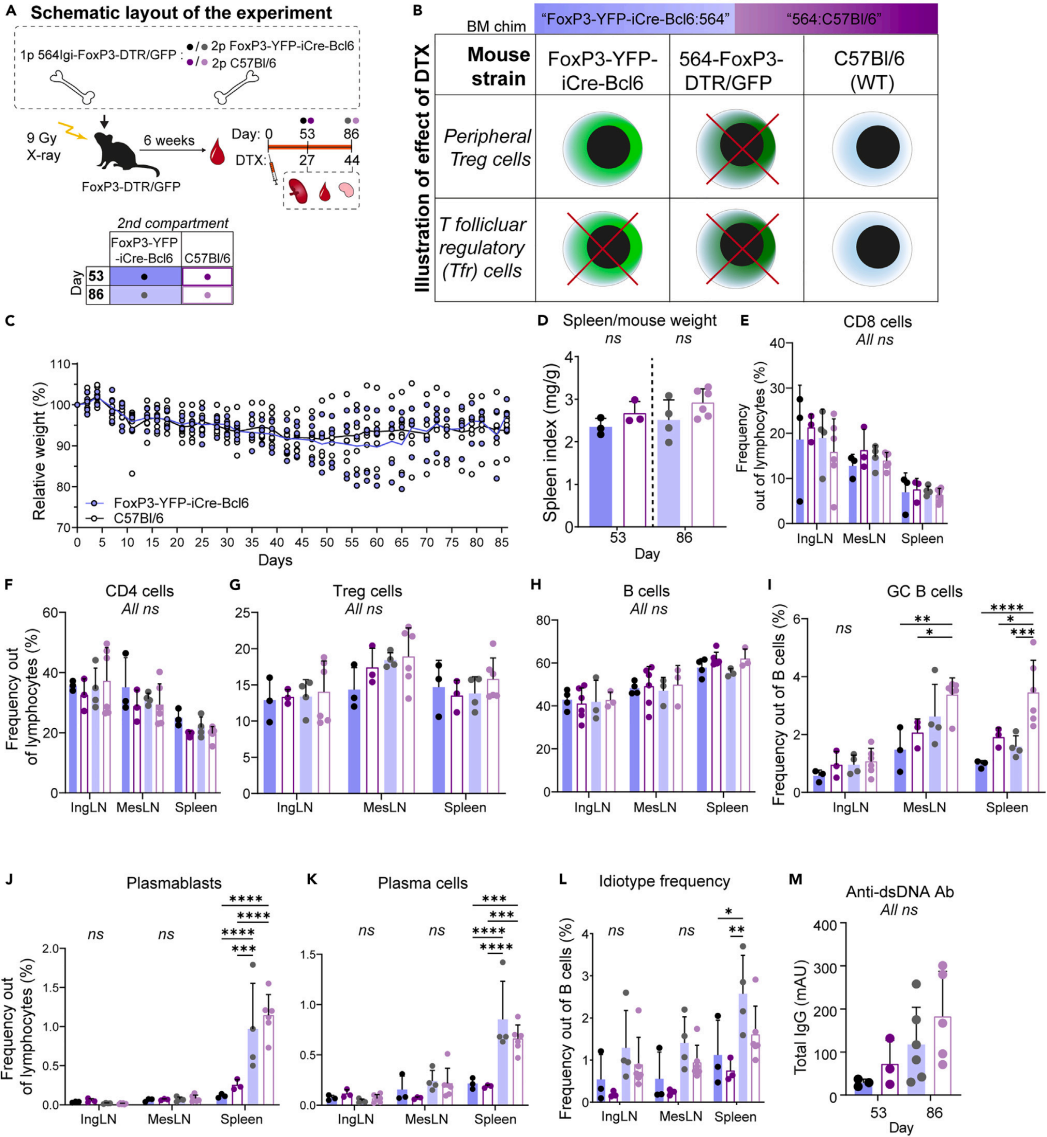
Long-term ablation of T-follicular regulatory cells (Tfr) does not exacerbate established autoimmunity (A) Schematic representation of the experimental layout. Chimeras were rested for 6 weeks, then bled to verify chimerism (Day 0), and DTX was injected every 3 days. Cohorts were analyzed at day 53 (27 DTX injections) or day 86 (44 DTX injections). (B) Schematic representation of the resulting ablation of the regulatory T cells upon the DTX treatment of the two bone marrow (BM) chimera groups. (C) Weight curves for the two groups relative to starting weight before diphtheria toxin (DTX) treatment. Circles indicate the weight of individual mice and curves represent the mean. (D) Spleen weight relative to total body weight. (E) Frequency of CD8 T cells in inguinal and mesenteric lymph nodes (IngLN and MesLN, respectively), and spleen for FoxP3-YFP-iCre-Bcl6 and C57BL/6J chimera groups at day 53 (27 DTX treatments, *n* = 3 and 3, respectively) and 86 (44 treatments DTX treatments, *n* = 4 and 6, respectively). (F) Frequency of CD4 T cells. (G) frequency of Treg cells. (H) Frequency of B cells. (I) Frequency of GC B cells. (J) Frequency of plasmablasts. (K) Frequency of plasma cells. (L) Frequency of idiotype positive B cells. (M) Levels of anti-dsDNA antibodies in serum. Bar graphs in (D–M) represent the mean ± SD and individual values are shown. Statistical significance was given for two-way ANOVA with Tukey’s posttest for (E–L) and one-way ANOVA with Tukey’s posttest in (D and M). Only significant comparisons are shown: ∗*p* < 0.05; ∗∗*p* < 0.01; ∗∗∗*p* < 0.001; ∗∗∗∗*p* < 0.0001, all other comparisons were not significant (ns, *p* > 0.05). In all instances, “days” refer to days from the first DTX injection. See also Figures S5 and S6.

We next considered the possibility that the time frame was too short to elicit a robust effect and instead asked how long-term ablation of Tfr would impact an ongoing autoreactive response. To this end, we again set up mixed bone marrow chimeras reconstituted with 564Igi FoxP3-GFP/DTR driver combined with the FoxP3-YFP-Cre Bcl6^flx/flx^ line, allowing the temporal ablation of Tfr specifically. However, instead of evaluating untreated chimeras as controls, we instead generated control chimeras reconstituted with 564Igi FoxP3-GFP/DTR driver combined with non-Tfr-blocked C57BL/6J bone marrow and treated these in parallel (Figure 2A). This should allow compensatory expansion of Tfr and should provide a better control for potential non-specific effects of DTX during the long treatment regimen (Figure 2B). We took blood samples from the chimeras 6 weeks post reconstitution and verified normal and comparable levels across main lymphocyte compartments (B cells, CD4 and CD8 T cells, Treg) (Figures S5A–S5D). At this time point, around 30% of Tregs in the FoxP3-YFP-iCre-Bcl6 chimeras were of YFP donor origin (Figure S5E), whereas in the B6 chimeras, a little over half were GFP negative (Figures S5F and S5G). We began DTX treatment and monitored the weight of the mice over the course of the treatment, revealing an initial drop of about 5% in both groups by day 10, after which the weight stabilized (Figure 2C). There were no significant differences in spleen weights relative to body weight (Figure 2D). CD8 T (Figure 2E, full gating strategy in Figure S6A), CD4 T (Figure 2F), and total Treg cell (Figure 2G) levels were comparable between the groups and time points. The frequency of B cells was comparable between the two groups at day 53 and day 86 post-treatment initiation (Figure 2H). GC B cells were present at robust levels in both groups at both time points, but somewhat surprisingly, at the late time point, GC B-cell levels were additionally elevated in the spleen of Tfr sufficient chimeras (Figure 2I). However, despite their lower GC B-cell frequency, the Tfr blocked chimeras displayed equally robust plasmablast (Figure 2J) and plasma cell (Figure 2K) levels in the spleen. This was also the case when plasmablast and plasma cell levels were normalized to B-cell frequencies (Figures S6B and S6C). The frequency of idiotype positive cells was elevated in the spleen of the Tfr blocked chimera group at the late time point (Figure 2L), but there were no significant differences in the levels of anti-dsDNA antibodies (Figure 2M). We verified that Tfr were robustly represented in the Tfr non-blocked chimeras, compared to the Tfr blocked controls (Figure S6D). Hence, in the chronic autoreactive scenario, Tfr appeared unsuccessful in controlling GCs and their plasma cell output, and hence ablating them had little effect.

### T-follicular regulatory cells increase in early-stage autoreactive germinal centers

**Figure 3:**
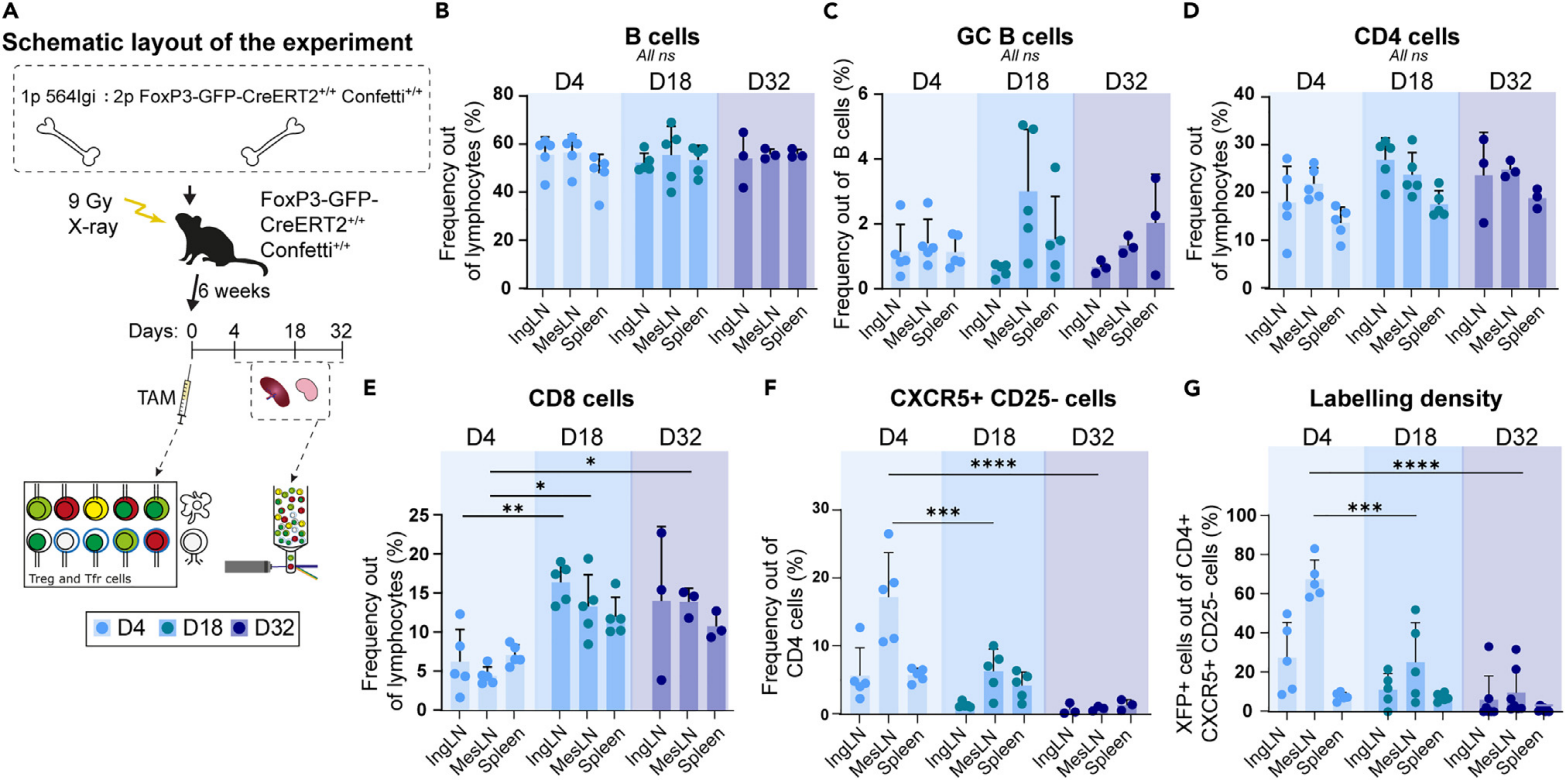
T-follicular regulatory cell (Tfr) clones induced in an autoimmune response are transient (A) Schematic representation of the experimental layout for the bone marrow (BM) chimera setup, where 564Igi BM cells were combined with BM cells from FoxP3-GFP-CreERT2^+/+^ Confetti^+/+^ mice and used to reconstitute lethally irradiated FoxP3-GFP-CreERT2^+/+^ Confetti^+/+^ recipients. After a reconstitution phase of 6 weeks, the mice were exposed to tamoxifen (TAM) to activate the confetti cassette in all FoxP3+ cells, leading to the expression of ten color combinations in peripheral T regulatory cells and Tfr (Day 0). Flow cytometry was performed at 4 (*n* = 5), 18 (*n* = 3) and 32 (*n* = 3) days post TAM administration. (B) Frequency of B cells. (C) Frequency of GC B cells. (D) Frequency of CD4 T cells. (E) Frequency of CD8 T cells. (F) Frequency of CXCR5+ CD25^−^ T cells. (G) Labeling density, determined as XFP+ cells out of CD4^+^ CXCR5+ CD25^−^ T cells. Bar graphs in (B–G) represent the mean +SD and individual values are shown. All-way comparisons were done using two-way ANOVA with Tukey’s post-test, but significance is given only for comparisons between time points per tissue. Significance is indicated as ns (not significant) *p* > 0.05; ∗*p* < 0.05; ∗∗*p* < 0.01; ∗∗∗*p* < 0.001; ∗∗∗∗*p* < 0.0001. In all instances, “days” refer to days from tamoxifen administration. See also Figure S7.

Our findings hitherto indicated an immediate impact of ablating Tfr, manifesting in an increased plasma cell output and relaxed idiotype positive cell frequency (Figures 1K and 1L). However, the autoreactive process subsequently caught up in non-ablated controls (Figures 1U and 1V), and in the long-term autoreactive scenario, Tfr sufficient mice fared no better than their Tfr ablated counterparts in terms of plasmablast, plasma cell, and anti-dsDNA antibody levels (Figure 2). Consequently, we asked the question what immediate adjustments were occurring in the Tfr compartment in connection with the induction of autoimmunity. We have previously characterized the kinetics of induction of the autoimmune response in the model, revealing that GCs form at some point between 4 and 6 weeks of reconstitution.^21^ To track the dynamics in the Tfr compartment, we sought to leverage the Confetti reporter previously used successfully to follow clonal expansion in the B-cell compartment.^19^^,^^29^^,^^30^ Accordingly, we crossed the FoxP3-GFP-CreERT2 line expressing Cre fused to a tamoxifen-regulated transactivator domain under the FoxP3 promotor. We again established bone marrow chimeras, this time combining 1 part 564Igi driver BM with 2 parts BM from our FoxP3-GFP-CreERT2^+/+^ Confetti^+/+^ line, into lethally irradiated FoxP3-GFP-CreERT2^+/+^ Confetti^+/+^ recipients. In this setup, all Tregs originating from two-thirds of the bone marrow and from the recipient side can be labeled temporally by the administration of the small-molecule estrogen analog tamoxifen (Figure 3A). We have previously established a robust protocol for the flow cytometric evaluation of labeling density and overall population distributions in FoxP3-GFP-CreERT2 and CD4-CreERT2 dependent Confetti lines.^31^ To home in on Tfr specifically, we combined our bone marrow chimera model with staining for CXCR5 and CD25, distinguishing the Tfr population (CXCR5+ CD25^−^) from the peripheral Treg population (CXCR5- CD25^+^). We pulsed the chimeras with tamoxifen by oral gavage at day 0, then analyzed the outcome at days 4, 18, and 32. B-cell and CD4 T-cell frequencies were comparable at the three time points (Figures 3B and 3D, gating strategy in Figure S7), whereas CD8 T-cell levels were a little lower at the first time point than at the two subsequent time points (Figure 3E), perhaps as a consequence of the BM reconstitution process. The mice presented with robust GC B-cell frequencies at all time points, across all tissues (Figure 3C). However, looking at CXCR5+ CD25^−^cells out of the CD4 T-cell gate, i.e., Tfr, there was a high frequency at the day 4 time point, which dropped markedly by 18 days and even further by 32 days (Figure 3F). This suggested that Tfr increase in early-stage autoreactive GCs, but are attenuated as these GC responses become chronic. We next examined the XFP labeling density among these Tfr, which by and large tracked with the overall population change (Figure 3G). That is, we temporally labeled a Tfr population around day 0 and saw a high degree of labeling at day 4, which subsequently waned by day 18 and even further by day 32.

### T-follicular regulatory cells induced in the autoimmune response are transient

**Figure 4:**
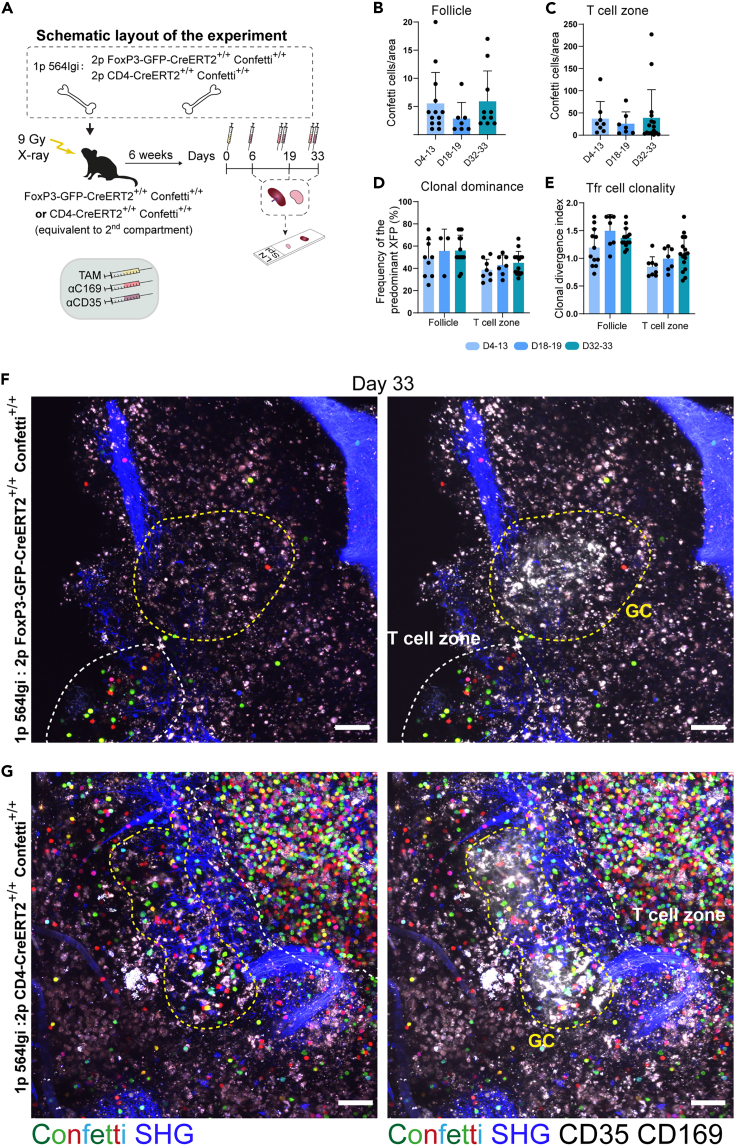
Autoreactive germinal centers harbor polyclonal T-follicular regulatory cell (Tfr) populations (A) Schematic representation of the experimental layout for the bone marrow (BM) chimera setup, where 564Igi BM cells were combined with either BM cells from FoxP3-GFP-CreERT2^+/+^ Confetti^+/+^ or CD4-CreERT2^+/+^ Confetti^+/+^ mice and used to reconstitute lethally irradiated recipients. After a reconstitution phase of 6 weeks, the mice were exposed to tamoxifen (TAM) to activate the confetti cassette in FoxP3+ or all CD4^+^ cells, for the respective groups (Day 0). Two days before the indicated analysis timepoints, mice received an i.p. injection of anti-CD35-A647, and 15 min before euthanasia they received anti-CD169-A647 i.v. The spleen and lymph nodes were imaged using two-photon microscopy and Confetti+ cells were quantified. (B) Quantification of Confetti+ cells in the follicle within three time intervals after TAM administration. (C) Quantification of Confetti+ cells in the T-cell zone within three time intervals after TAM administration. (D) Clonal dominance, i.e., frequency of the most dominant XFP in the follicle or T-cell zone within the three time intervals. (E) Clonal divergence index, i.e., distance from random XFP distribution in the follicle or T-cell zone within the three time intervals. Bar graphs in B-E represent the mean +SD and individual values are shown. (F) Representative two-photon micrograph from the spleen of a FoxP3 Confetti BM chimera at Day 33 post tamoxifen, showing Confetti colors (multicolor) and collagen second harmonic generation (SHG, blue), without (left) or with (right) CD35 and CD169 labeling (far-red signal in a separate channel, represented in white in the micrographs). The broken yellow line indicates the margin of the follicle/germinal center, and the broken white line indicates the T-cell zone. (G) Representative two-photon micrograph from the spleen of a CD4 Confetti BM chimera for comparison. Additional representative examples of spleen and lymph node micrographs can be found in Figures S8 and S9 for FoxP3 and CD4 BM chimeras, respectively. All quantification was performed on spleen *ex vivo* samples and data in (B–E) represent nine follicles from four mice (day 4–13), six follicles from three mice (day 18–19), and sixteen follicles from four mice (day 32–33). Presented micrographs were color-adjusted for visual clarity. Scale bars in (F) and (G) represent 50 μm. In all instances, “days” refer to days from tamoxifen administration. See also Figures S8 and S9.

We next sought to quantify what was going on at a clonal level within this Tfr population. To this end, we combined our BM chimera setup with intravital labeling and readout by two-photon microscopy of spleen and lymph node explants (Figure 4A). Because of an expected low number of Confetti+ cells in the FoxP3 reporter, we also generated in parallel total CD4-CreERT2 reporter chimeras. We used anti-CD169 (MOMA-1) to label the metallophilic macrophages of the marginal zone and anti-CD35 (CR1) to label the follicular dendritic cell network of the follicle, and additionally relied on second-harmonics signal from structural collagen for anatomical context, allowing us to define follicular B-cell versus T-cell zones associated with the periarteriolar lymphoid sheath (PALS). Quantifying Confetti cells (XFP+) within these areas revealed low but appreciable numbers of Tfr in the follicle (Figure 4B), and more significant numbers of Tregs in the T-cell zone (Figure 4C). Looking at the frequency of the most predominant color and the clonal divergence index, it was clear that these also remained constant in both anatomical locations (Figures 4D and 4E). Both parameters were slightly higher in the Tfr population of the follicle than in the T-cell zone, however, this was likely an effect of the very low number of observed cells, skewing toward higher color dominance and higher divergence from baseline. Nonetheless, despite the low number of Confetti+ cells in the FoxP3 driver chimeras, we were able to faithfully assign all color combinations, as also verified by our control group of CD4 driver chimeras (Figures 4F, 4G, S8, and S9).

### Partial T-follicular regulatory cell ablation is compensated by polyclonal T-follicular regulatory cell expansion

**Figure 5:**
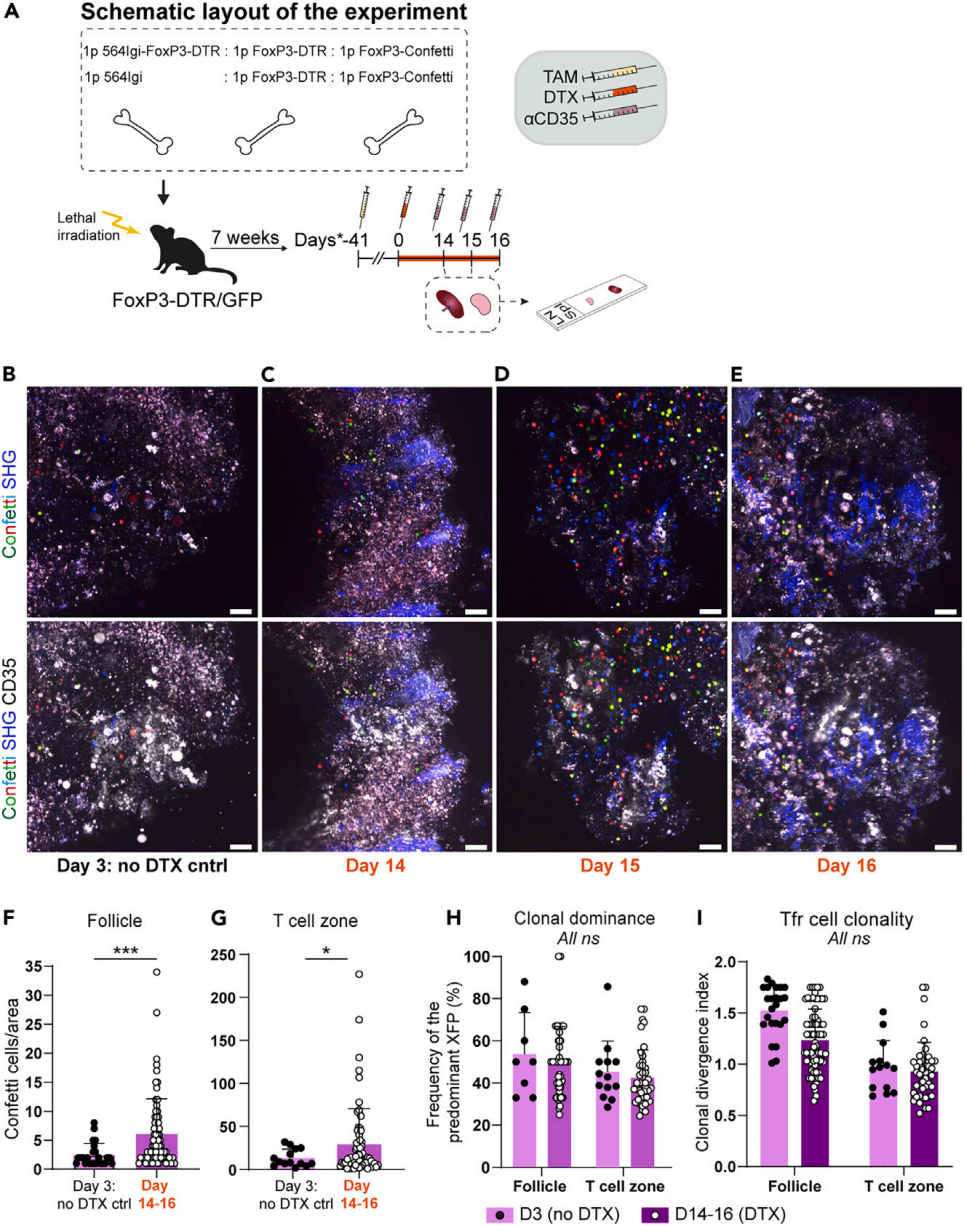
Partial ablation of the Treg population causes a polyclonal expansion in the remaining Treg population (A) Schematic representation of the experimental layout for the bone marrow (BM) chimera setup, where 1 part (p) 564Igi or 564Igi-FoxP3-DTR BM cells were combined with 1p FoxP3-DTR and 1p FoxP3-GFP-CreERT2^+/+^ Confetti^+/+^ BM and used to reconstitute lethally irradiated FoxP3-DTR recipients. After a reconstitution phase of 7 weeks, the mice were exposed to tamoxifen (TAM) to activate the Confetti cassette in FoxP3+ cells. Mice were subsequently DTX treated every three days (from Day 0) to ablate FoxP3-DTR-derived Tfrs. Two days before the indicated analysis timepoints, mice received an i.p. injection of anti-CD35-A647, and in some cases 15 min before euthanasia they received anti-CD169-A647 i.v. (B) Representative micrograph of spleen from a non-depleted control chimera, showing Confetti colors (multicolor) and collagen second harmonic generation (SHG, blue), without (top) or with (bottom) CD35 labeling (far-red signal in a separate channel, represented in white in the micrographs). (C) Representative micrograph of spleen from a Treg-depleted chimera at day 14 post DTX treatment start. (D) As C, but for day 15. (E) As C, but for day 16. Scale bars in (B–E) represent 50 μm. (F) Quantification of Confetti+ cells in the follicle at day 3 (no DTX) vs. day 14–16 post DTX. (G) Quantification of Confetti+ cells in the T-cell zone at day 3 (no DTX) vs. day 14–16 post DTX. (H) Clonal dominance, i.e., frequency of the most dominant XFP in the follicle or T-cell zone at day 3 (no DTX) vs. day 14–16 post DTX. (I) Clonal divergence index, i.e., distance from random XFP distribution in the follicle or T-cell zone at day 3 (no DTX) vs. day 14–16 post DTX. Day 3 data are derived from two mice and 26 follicles counted in total, day 14–16 data are from five mice and 84 follicles counted in total. Bar graphs in (F–I) represent the mean +SD, and individual values are shown. Significance given for Mann-Whitney t-test, ns (not significant) *p* > 0.05; ∗*p* < 0.05; ∗∗∗*p* < 0.001. In all instances, “days” refer to days from DTX administration.

Having established that Tfr were present in the autoreactive GCs, but overall, they began to wane in the chronic GC response, we asked whether Tfr had the capacity to expand in a scenario where part of the population was ablated. Again, we relied on our bone marrow chimera model, establishing chimeras in which either 1 part 564Igi or 564Igi-FoxP3-DTR driver marrow was combined with 1 part FoxP3-DTR and 1 part FoxP3-Confetti bone marrow, into lethally irradiated FoxP3-DTR/GFP recipients (Figure 5A). In this scenario, one-third of the Tregs could be labeled in the Confetti colors, and one or two-thirds of Tregs could be ablated by the administration of DTX. We again read the behavior of the Confetti+ cells using intravital labeling followed by two-photon imaging of explants, starting at day 3 in untreated controls (Figure 5B), and through days 14 (Figure 5C), 15 (Figure 5D) and 16 (Figure 5E) post tamoxifen. Quantifying Confetti+ cells in the follicle at the early non-treated time-point revealed a low, but appreciable number of Tfr (Figure 5F), congruent with that observed previously (Figure 4B), when taking into account that a lower fraction of the Treg population carried the reporter in this chimera setup. Upon the DTX-mediated ablation of non-Confetti Tregs, however, this fraction was seen to increase significantly (Figure 5F), and this effect was more marked than in the T-cell zone (Figure 5G). This demonstrated a homeostatic expansion of both these subsets in the face of a reduction in their frequency, suggesting that they were actively responding in the autoreactive scenario. Furthermore, looking at the clonal dominance (Figure 5H) and clonal divergence index (Figure 5I), there was no difference between treated and untreated mice across the two zones, demonstrating that both Tfr and Tregs were undergoing polyclonal expansion.

## Discussion

Tfr have been demonstrated to prevent the outgrowth of self-reactive B-cell clones following T-dependent antigen immunization,^11^ to limit the expansion of self-reactive B cells in connection with influenza infection,^32^ to control humoral and allergic immunity by restraining early B-cell responses,^33^ and to control autoimmunity.^15^ However, most physiologically relevant models of autoimmune disease occur in the presence of Tfr, raising the question of whether Tfr are truly able to restrain autoreactive responses.

Here, in a model of established autoimmunity, we found that the depletion of Tfr did not alter the frequency of GC B cells (Figures 1I and 1T); however, we did observe a relaxed early output of plasma cells in the spleen (Figure 1K). Tfr depletion also resulted in a slightly lower frequency of B cells carrying the idiotypic receptor (Figure 1L), potentially suggesting increased somatic hypermutation (resulting in affinity maturation toward autoantigen, but loss of anti-idiotypic reactivity). However, taken together, this at best indicated a mildly relaxed control of plasma cell output and autoreactive B-cell selection in the spleen upon the timed ablation of Tfr. Somewhat surprisingly, long-term ablation of Tfr also did not exacerbate established autoimmunity (Figure 2). Curiously, at the late time point, GC B-cell levels were in fact additionally elevated in the spleen of Tfr sufficient chimeras (Figure 2I). Indeed, Tfr have previously been shown to support GC responses and B-cell affinity maturation in some models of viral infection, immunization, and allergy.^34^^,^^35^^,^^36^^,^^37^ Hence, it appears that Tfr play a multifaceted role in the fine-tuning of the GC response. Mechanistically, they negatively regulate GC B cells that react to autoantigen, whereas they promote the centroblast transcriptional program of foreign-antigen directed GC B cells via IL-10.^35^^,^^37^ Intriguingly, targeting of nuclear proteins to antigen-specific B cells triggers the rapid accumulation of Tfr with immunosuppressive characteristics.^38^ Our model is driven by nuclear autoantigen-reactive B cells,^39^ displays epitope spreading to additional nuclear targets, and exhibits demonstrable autoreactivity in at least 4 out of 5 GCs.^19^^,^^21^ Consequently, our interpretation is that in the chronic autoreactive scenario, Tfr are unsuccessful in controlling GCs and their plasma cell output, and hence ablating them has little effect. This is in line with the notion of Sage, Sharpe, and colleagues that Tfr regulate early, but not late, GC responses to control antigen-specific antibody and B-cell memory.^33^ Our findings using the FoxP3-Confetti reporter setup lends further support to this interpretation because we find that Tfr clones induced in the autoimmune response expand transiently, highlighting their inability to contain the chronic response (Figure 3). Early work from the group of Luis Graca in a foreign-antigen immunization setting showed that Tfr proliferated during GC expansion, from day 4 to day 12. Of note, here we are not able to observe the very early expansion, because the GC response is initiated sometime between weeks 4 and 6,^21^ and we only label Tfr subsequent to this point, at a time when the autoreactive response is becoming established. The advantage of this approach, however, is that we catch a larger fraction of Tfr with our labeling strategy. Interestingly, while global analysis of such a pulsed population of Tfr indicated a significant turnover (Figure 3G), on an individual GC level, Tfr in our chronic autoreactive GCs appeared stably polyclonal throughout the window of investigation from day 4 to day 33, based on color dominance and clonal divergence index measurements (Figures 4D and 4E). This may be reconciled by the notion that some GCs over time “go dark,” i.e., some GCs become dominated by unlabeled Tfrs, whereas others remain labeled. On average, this would lead to a decrease in Tfr labeling density over time, whereas on an individual GC basis, we do not observe the dark GCs, and hence the number of Tfrs in labeled GCs could remain more consistent over the investigated time frame. This may be further compounded by subtle differences in Tfr definition between the flow cytometric and imaging readouts. In the former, we rely on a relatively simple marker strategy of CD4^+^CD25-CXCR5+, whereas in the latter we base it on localization in the follicle. Since CXCR5+ is not itself necessary/sufficient for follicular localization^40^ and circulating Tfr can also be found,^10^ we may be picking up additional Tfr in the flow analyses, which have left the GC and are recirculating. Finally, it is not known if autoreactive GCs are themselves chronic, i.e., the same GC persists over very long periods of time, or whether individual GCs are “dying” and new ones being “born” over the course of the autoreactive response. Such considerations notwithstanding, our results suggest that Tfr do not undergo significant clonal selection and expansion during the course of the chronic response, something which might contribute to explaining their inability to suppress the autoimmune response. However, an alternative explanation, which we cannot rule out based on our data, is that a given Tfr clone is not restricted to its specific follicle but can freely (re)distribute between neighboring follicles, which would mask local clonal expansion. Of note, Tfh have previously been reported to distribute freely, lending some credence to this possibility,^41^ although it remains unclear whether Tfr behave in a similar manner. Interestingly, when we perturbed the homeostatic conditions by the partial ablation of the Treg population, this resulted in a polyclonal expansion in the remaining Tfr population (Figure 5F) similar to that of conventional Tregs (Figure 5G). Again, this appeared to be polyclonal in nature, based on color dominance and clonal divergence index measurements (Figures 5H and 5I, respectively), albeit again with the caveat that expanded Tfr clones might redistribute, thereby obscuring oligoclonal expansions. Nonetheless, the expansion response upon perturbation suggests that overall Tfr levels are somehow homeostatically controlled, yet they are still incapable of curbing the chronic autoimmune response.

The very basis of defining Tfr has recently been cast into doubt based on the observation by Victora and colleagues that Tfh in end-stage GCs upregulate FoxP3 to shut down the GC response.^16^ Already early on, Linterman, Vinuesa, and colleagues considered whether Tfr could represent induced Treg cells that arise from Tfh which switch on FoxP3 in the GC, as opposed to deriving from FoxP3+ Treg cells.^11^ Using adoptive transfers, they showed that 3A9 TCR^HEL^ transgenic T cells and OT-II transgenic T cells could form Tfh, but not Tfr. Furthermore, adoptively transferred FoxP3+ T cells retained FoxP3 expression and could form Tfr, whereas FoxP3- T cells could not. This was recapitulated with thymic CD4 single-positive cells expressing vs. not expressing FoxP3. Finally, the ablation of all peripheral Treg prior to immunization precluded the development of Tfr in an immunization setting.^11^ This was supported by independent findings of Chen Dong’s group, showing that Tfr derived from CXCR5- Treg cells and that Tfr by-and-large expressed the transcription factor Helios indicating nTreg origin,^12^ as well as work by Luis Graca and colleagues showing that Tfr derive from natural Tregs, whereas Tfh cells are not amenable to TGF-beta dependent conversion into FoxP3+ regulatory cells.^13^ Later work by the group of André Ballesteros-Tato demonstrated that high IL-2 levels during the peak of an influenza virus infection prevented the development of Tfr, but that concomitant with the resolution of the infection, Treg cells downregulated CD25, upregulated Bcl-6, and differentiated into Tfr, which subsequently prevented the expansion of autoreactive B cells.^32^ Indeed, using adoptive transfers, Ballesteros-Tato and colleagues also noted that some CD4+FoxP3- cells upregulated FoxP3 after infection. However, contrary to the findings of Victora et al., and in line with Linterman, Vinuesa, Chen, and Graca, they concluded that true Tfr were only generated from CD25hiFoxP3+ precursors. In support of the existence of two differential origins of Tfr in mice, two distinct lineages of Tfr were recently found in human tonsils.^42^ One of these subsets, nTfr, was clonally related to nTreg, and the other, iTfr, was found to be likely induced by Tfh cells. The former was found to be elite suppressors localized in follicular mantles, whereas the latter were found to gain suppressive function while retaining the capacity to help B cells.^42^

On a conceptual note, there is a strong argument for why Tfr should exist: although the requirement for linked recognition should be sufficient to ensure divergence from the eliciting antigen in the GC process, central tolerance of the T-cell repertoire is not stringent. Therefore, Tfh can come into existence which support autoreactive GC B-cell selection, and this necessitates a dominant-negative mechanism.

Our findings in the FoxP3-Cre Bcl6^flx/flx^ autoimmune model agree well with those of Ballesteros-Tato et al. in an influenza virus infection setting,^32^ where they observed normal accumulation of Tfh and GC B cells, whereas the CD138+ cells were increased, suggesting that lack of Tfr does not change Tfh or GC B-cell responses, but instead promotes the accumulation of CD138+ antibody secreting cells.

In conclusion, here we found that Tfr were unable to control an ongoing autoreactive response. Ablation caused an acute relaxation in the control of plasma cell output, but no long-term effects could be observed. Our data furthermore demonstrated a polyclonal expansion of Tfr in the autoimmune setting. Taken together, our findings suggest that Tfr can restrict early GC responses but are dysfunctional in chronic autoimmunity, raising the question of whether restoring Tfr function can limit autoimmune progression.

### Limitations of the study

While our study sheds light on the role of Tfr in an acute autoreactive response and chronic, systemic autoimmunity, several limitations warrant consideration. We have relied on transgenic color-encoding for Tfr clonality assessment, but this could be strengthened by TCR sequencing. Future research would benefit from an extension to additional models of systemic autoimmunity to demonstrate the generalizability of our findings. Additionally, further investigation is warranted to elucidate how Tfr behave in autoimmune responses directed at limited or tissue-restricted autoantigens.

## Resource availability

### Lead contact

Further information and requests should be directed to and will be fulfilled by the lead contact, Søren E. Degn (sdegn@biomed.au.dk).

### Materials availability

This study did not generate new unique reagents.

### Data and code availability

This study did not generate standardized datasets. All data reported in this article will be shared by the lead contact upon request. This article does not report original code. Any additional information required to reanalyze the data reported in this article is available from the lead contact upon request.

## Acknowledgments

We would like to thank the FACS Core and the BioImaging Core at AU for assistance with flow cytometry and imaging, respectively.

This work was supported by the 10.13039/501100012331LEO Foundation (to Søren E. Degn, grant ID: LF-OC-22-000977), 10.13039/501100003554Lundbeckfonden (to Cecilia Fahlquist-Hagert, grant ID: R303-2018-3415), 10.13039/501100009708Novo Nordisk Fonden (to Søren E. Degn, grant ID: NNF17OC0028160), and the Independent Research Fund Denmark (to Søren E. Degn, Grant ID: 9060-00038).

## Author contributions

Conceptualization, C.F-H., T.R.W., and S.E.D.; methodology, C.F-H., T.R.W., and S.E.D.; investigation, C.F-H., T.R.W., M.K.P., and L.J.; writing – original draft, C.F-H. and S.E.D.; writing – review and editing, C.F-H., T.R.W., and S.E.D.; visualization, C.F-H. and S.E.D.; supervision, S.E.D.; project administration, S.E.D.; funding acquisition, C.F-H. and S.E.D.

## Declaration of interests

The authors declare that they have no conflicts of interest in relation to the presented work.

## STAR★Methods

### Key resources table

**Table:** 

REAGENT or RESOURCE	SOURCE	IDENTIFIER
**Antibodies**		

Anti-564Igi idiotype, clone 9D11	Chatterjee et al.^43^	N/A
Anti-B220-A700, clone RA3-6B2	BD Biosciences	Cat#557957; RRID: AB_396957
Anti-B220-BV510, clone RA3-6B2	BD Biosciences	Cat#563103; RRID: AB_2738007
Anti-B220-PB, clone RA3-6B2	BD Biosciences	Cat#558108; RRID: AB_397031
Anti-CD138-BV650, clone 281-2	BD Biosciences	Cat#564068; RRID: AB_2738574
Anti-CD38-PE-Cy7, clone 90	BioLegend	Cat#102718; RRID: AB_2275531
Anti-CD4-PerCP, clone RM4-5	Biolegend	Cat#100538; RRID: AB_893325
Anti-CD4-Qdot605, clone RM4-5	ThermoFisher Scientific	Cat#Q10092; RRID: AB_10374736
Anti-CD8-PerCP-Cy5.5, clone SK1	BD Biosciences	Cat#565310; RRID: AB_2687497
Anti-FoxP3-PE-eFluor610, clone FJK-16s	Thermo Fisher Scientific	Cat#61-5773-82; RRID: AB_2574624
Anti-FoxP3-PE, clone FJK-16s	Thermo Fisher Scientific	Cat#72-5775-40; RRID: AB_469978
eBioscience™ Fixable Viability Dye - eFlour780	Thermo Fisher Scientific	Cat#65–0865-14
Anti-CD35, clone 8C12	BD Biosciences	Cat#553816; RRID: AB_395068
Anti-CD169, clone 3D6.112	BioLegend	Cat#142407; RRID: AB_2563620
Anti-CXCR5-biotin, clone 2G8	BD Biosciences	Cat#551960; RRID: AB_394301
Streptavidin-BV421	BD Biosciences	Cat#563259
Streptavidin-BV785	BD Biosciences	Cat#563858
Anti-CD25-BV786, clone PC61	BD Biosciences	Cat#564023; RRID: AB_2738548
Anti-CD95-PE, clone Jo2	BD Biosciences	Cat#554258; RRID: AB_395330
Anti-CD95, clone Jo2	BD Biosciences	Cat#554254; RRID: AB_395326
Biotinylated goat-*anti*-mouse Ig	Southern Biotech	Cat#1010-08
Fc-block	BD Biosciences	Cat#553142; RRID: AB_394657
Eu3+-labelled streptavidin	PerkinElmer	Cat#1244-360

**Chemicals, peptides, and recombinant proteins**		

Salmon sperm dsDNA	ThermoFisher Scientific	AM9680
Bovine serum albumin (BSA)	Sigma-Aldrich	A4503
Fetal calf serum (FCS)	Life Technologies	Cat#10270106
PBS	Lonza	Cat#BE17-515Q
NH_4_Cl	Merck	Cat#1.01145.0500
NaHCO_3_	Merck	Cat#1.06329.0500
Diphtheria toxin	Sigma-Aldrich	Cat#D0564-1MG
Lympholyte-M Cell Separation Media	Cedarlane labs	Cat#CL5030
Formalin	Sigma-Aldrich	Cat#F1635
TBS	Fisher Scientific	Cat#10467583
Amoxicillin	ScanVet	Cat#531194
Enhancement buffer	Amplicon	Cat#AMPQ99800
Tween 20	Merck	Cat#8.17072.1000
ViaStain AOPI staining solution	Nexcelom Bioscience	Cat#CS2-0106-25mL
Tamoxifen	Sigma-Aldrich	Cat#T5648-5g
Sunflower oil	Sigma-Aldrich	Cat#S5007-1L
iFluor® 647 succinimidyl ester	AAT Bioquest	Cat#1031
EDTA	Merck	Cat#1.08418.0250

**Critical commercial assays**		

eBioscienceTM Foxp3/Transcription Factor Staining Buffer Set	ThermoFisher Scientific	Cat#00-5523-00

**Experimental models: organisms/strains**		

Mouse: B6.129(Cg)-FoxP3^tm4(YFP/icre)Ayr^/J	Jackson Laboratories	RRID:IMSR_JAX:016959
Mouse: B6.129S(FVB)-Bcl6^tm1.1Dent^/J	Jackson Laboratories	RRID:IMSR_JAX:023727
Mouse: B6.129S4(Cg)-*Igk*^*tm1(Igk564)Tik*^*Igh*^*tm1(Igh564)Tik*^/J	Jackson Laboratories	RRID:IMSR_JAX:032723
Mouse: B6(129X1)Tg(Cd4cre/ERT2)11Gnri/J	Jackson Laboratories	RRID:IMSR_JAX:022356
Mouse: B6.129P2-*Gt(ROSA)26Sor*^*tm1(CAGBrainbow2.1)Cle*^/J	Jackson Laboratories	RRID:IMSR_JAX:017492
Mouse: *Foxp3*^*tm9(EGFP/cre/ERT2)Ayr*^/J	Jackson Laboratories	RRID:IMSR_JAX:016961
Mouse: C57BL/6JRj	Janvier Labs	2017-01-ENG-RM-20

**Software and algorithms**		

FlowJo^TM^ version 10.8.1	BD	https://www.flowjo.com/solutions/flowjo/downloads
GraphPad Prism v. 9.	GraphPad Software	https://www.graphpad.com/
BD FACSDiva v. 8.0.2	BD	https://www.bdbiosciences.com/en-us/products/software/instrument-software/bd-facsdiva-software
NovoExpress v. 1.6.2	Agilent	https://www.agilent.com/en/product/research-flow-cytometry/flow-cytometry-software/novocyte-novoexpress-software-1320805
Fiji, ImageJ v. 2.14.0/1.54f	Schindelin et al., 2012^44^	https://imagej.net/software/fiji/downloads

**Other**		

Pestles	Argos Technologies	Cat#7339-901
Microtiter plates (FluoroNunc Maxisorb)	Thermo Scientific	Cat#437796

### Experimental model and study participant details

#### Ethics statement

All animal experiments were conducted in accordance with the guidelines of the European Community and were approved by the Danish Animal Experiments Inspectorate (protocol numbers 2017-15-0201-01348 and 2017-15-0201-01319).

#### Experimental model and subject details

B6.129(Cg)-*FoxP3*^*tm4(YFP/icre)Ayr*^/J^24^ and B6.129S(FVB)-*Bcl6*^*tm1.1Dent*^/J^25^ were purchased from Jackson laboratories (JAX stock #016959 and #023727, respectively) and intercrossed. FoxP3-YFP-iCre Bcl6^flx/flx^ mice used in experiments were Cre homozygous (females) or hemizygous (males) and Bcl6^flx^ homozygous. The 564Igi line (B6.129S4(Cg)-*Igk*^*tm1(Igk564)Tik*^ *Igh*^*tm1(Igh564)Tik*^/J)^39^ was kindly made available by Thereza Imanishi-Kari, Tufts University, and provided by Michael C. Carroll, Boston Children’s Hospital. B6(129X1)Tg(Cd4cre/ERT2)11Gnri/J (JAX stock #022356) was crossed to B6.129P2-*Gt(ROSA)26Sor*^*tm1(CAGBrainbow2.1)Cle*^/J (JAX stock #017492). CD4-CreERT2-Confetti mice used in experiments were Cre hemizygous and Confetti homozygous. Similarly, *Foxp3*^*tm9(EGFP/cre/ERT2)Ayr*^/J (JAX stock #016961) was crossed to B6.129P2-*Gt(ROSA)26Sor*^*tm1(CAGBrainbow2.1)Cle*^/J. FoxP3-GFP-CreERT2-Confetti mice used in experiments were Cre homozygous (females) or hemizygous (males) and Confetti homozygous. Wild-type C57BL/6JRj mice were purchased from Janvier Labs. Mice were housed in the Animal Facility at the Department of Biomedicine, Aarhus University, Denmark, under specific pathogen-free (SPF) conditions in individually ventilated cages at ambient temperature of 20°C–22°C and ambient humidity, on a 12-h light/dark cycle with standard chow and water *ad libitum*. Both female and male mice were used throughout, with balanced representation across groups to account for weight and sex differences. BM recipients were 8–18 weeks old at irradiation and donors were 7–22 weeks old at time of BM harvest.

### Method details

#### Bone marrow chimeras

Recipient mice were irradiated with 9 Gy (internal dosimetry) in a MultiRad 350 (Faxitron), with 350 kV, 11.4 mA, a Thoraeus filter [0.75 mm Tin (Sn), 0.25 mm Copper (Cu), and 1.5 mm Aluminum (Al)], and with a beam-distance of 37 cm.^19^^,^^45^ Irradiated recipients were kept on antibiotic water (0.25 mg amoxicillin (531194, ScanVet) per mL drinking water) to avoid opportunistic infections. On the following day, donor mice were anesthetized with continuous flow of 3% isoflurane and euthanized by cervical dislocation. Femora, fibulae/tibiae, ossa coxae and humeri were harvested, mechanically cleaned, and rinsed in FC buffer (PBS (BE17-515Q, Lonza), containing 2% heat-inactivated fetal calf serum (FCS, 10270106, Life Technologies), and 1 mM ethylenediaminetetraacetic acid (EDTA, 1.08418.0250, Merck)). The bones were crushed in a mortar to release the bone marrow (BM) cells, and the cell extract was then passed through a 70 μm cell strainer. The donor BM cells were counted in a Cellometer K2 cell counter (Nexcelom) using Acridine orange (AO) and propidium iodide (PI) (ViaStain AOPI staining solution, CS2-0106-25mL, Nexcelom Bioscience). Cells from the desired combinations of mice were then mixed according to the proportions mentioned in the figure legends, pelleted by centrifugation (200 *g*, 10 min, 4°C) and resuspended to 1∗10^8^ cells/mL. The donor cell mixtures were used to reconstitute the recipient mice by retroorbital injection of 200 μL (containing a total of 20∗10^6^ cells) into each recipient mouse. Cohorts were analyzed at the times post reconstitution indicated in the figure legends.

#### Tissue harvest and preparation

Following anesthesia in continuous flow of 3% isoflurane, blood was collected from the retroorbital plexus via a microcapillary tube into Eppendorf tubes containing 200 μL PBS with 5 mM EDTA. At endpoint, mice were anesthetized and either bled retroorbitally followed by cervical dislocation, or mice were decapitated followed by blood collection. Spleen, inguinal and mesenteric lymph nodes (IngLN and MesLN, respectively) were harvested, and the spleen was divided using surgical scissors. Tissues were either placed into ice-cold FC buffer for flow cytometry analysis or prepared for two-photon analysis (see later in discussion). For flow cytometry, spleen and lymph nodes in ice-cold FC buffer were mechanically dissociated using pestles (7339-901, Argos Technologies). Samples were filtered through 70 μm cell strainers. Spleen samples were centrifuged at 200 *g* for 5 min at 4°C, lysed in RBC lysis buffer (155 mM NH_4_Cl (1.01145.0500, Merck), 12 mM NaHCO_3_ (1.06329.0500, Merck), 0.1 mM EDTA), incubated at RT for 3 min, centrifuged, and finally resuspended in FC buffer.

#### Flow cytometry

Twenty μL Fc-block (553142, BD Biosciences) diluted 1:50 in FC buffer and 100 μL of each sample was added onto a 96-well plate then incubated for 5–10 min on ice. Antibodies and fixable viability dye (65-0865-14, ThermoFisher Scientific) were diluted 1/300-1/500 and 1/2000, respectively, in FC buffer. The hybridoma producing anti-564Igi idiotypic antibody, clone 9D11,^43^ was kindly provided by Elisabeth Alicot, Boston Children’s Hospital, and produced in-house. All other antibodies were commercially available (see key resources table). One hundred μL antibody mix was added to each sample well and incubated for 30 min on ice. The plate was centrifuged at 200 *g* for 5 min, supernatant was removed, and cells were fixed for 30 min in PBS, 0.9% formaldehyde (F1635, Sigma-Aldrich) at RT. Following fixation, the plates were centrifuged at 200 *g* for 5 min, the supernatant discarded, and the samples resuspended in FC buffer. For CXCR5 staining, samples were incubated at 37°C with anti-CXCR5-biotin for 30 min, then washed, before regular staining on ice with streptavidin-BV421 and other cell-surface markers. For intracellular staining for FoxP3, after cell-surface marker staining cells were fixed and permeabilized using the eBioscience Foxp3/Transcription Factor Staining Buffer Set (00-5523-00, ThermoFisher), then incubated with anti-FoxP3-PE or PE-eFluor510. Flow cytometry evaluation was performed using either a LSRFortessa equipped with 4-laser (405 nm, 488 nm, 561 nm, 640 nm) and 16 fluorescence detectors (BD Biosciences, San Jose, CA) or a Novocyte Quanteon 4025 equipped with 4 lasers (405 nm, 488 nm, 561 nm and 637 nm) and 25 fluorescence detectors (Agilent, Santa Clara, CA). Data were acquired in either BD FACSDiva Software version 8.0.2 (LSRFortessa, BD Biosciences, San Jose, CA) or NovoExpress version 1.6.2 (Quanteon, Agilent, Santa Clara, CA) and analyzed in FlowJo version 10.8.1 software (BD).

#### Diphtheria toxin treatment

Mice were anesthetized with isoflurane as previously, and received 1 μg, 0.5 μg, or 0.25 μg (for mice with starting weights of ∼25–30 g, corresponding to around 33–40, 17–20, and 8–10 μg/kg, respectively) DTX (D0564-1MG, Sigma-Aldrich) in PBS by i.p. injection.

#### Tamoxifen treatment

To activate the Confetti cassette, 0.5 mL of tamoxifen (T5648-5g, Sigma-Aldrich) dissolved in sunflower oil (S5007-1L, Sigma-Aldrich) at a concentration of 30 mg/mL was administered by a single gavage for a total dose of 15 mg per mouse.

#### Intravital labeling

Two days before sacrifice and explant imaging, mice were briefly anesthetized with isoflurane as before, and received an i.p. injection of 5 μg anti-CD35 (clone 8C12, 553816, BD Pharmingen) labeled in-house using iFluor 647 succinimidyl ester (1031, AAT Bioquest) in sterile PBS. In some cases, 15 min before euthanasia for analysis, mice were similarly anesthetized and received 2 μg anti-CD169-A647 (MOMA-1, clone 3D6.112, 142407, BioLegend) i.v.

#### Two-photon microscopy

Mice were euthanized by cervical dislocation at the indicated time-points after tamoxifen injection, and spleen, IngLN, and MesLN were harvested as described above. The detailed protocol for imaging chamber preparation and two-photon imaging was as previously described.^46^ In brief, an imaging chamber was prepared to accommodate whole IngLN, MesLN and spleen sections of 2–3 mm thickness and imaged using an Olympus FVMPE-RS multiphoton Laser Scanning microscope, fitted with a 25×1.05 NA water-immersion objective, a MaiTai (e)HP DeepSee Ti-Sapphire laser (690–1040 nm from Spectraphysics), and 4 detectors (2 cooled GaAsP, and 2 regular alkali PMTs). Imaging of Confetti alleles was performed using an excitation wavelength of 940 nm. Fluorescence emission was recorded in 4 channels (mCFP/nGFP, 460–500 nm; nGFP/YFP, 520–560 nm; RFP, 535–610 nm; and CD35-A647, 620–680). For each follicle, we analyzed 25–34 optical sections with a step size of 4 μm, covering a total z-thickness of 94–136 μm/follicle. Data analysis was performed in Fiji, ImageJ v. 2.14.0/1.54f.^44^

#### Anti-dsDNA measurements by time-resolved immunofluorometric assay (TRIFMA)

FluoroNunc Maxisorp 96-well plates (437796, Thermo Scientific) were coated with 100 μg/mL salmon sperm dsDNA (AM9680, ThermoFisher Scientific) in PBS and incubated overnight at 4°C. Wells were blocked with 200 μL TBS (10467583, Fisher Scientific) containing 1% bovine serum albumin (BSA) (A4503, Sigma-Aldrich) for 1 h at RT and washed three times with TBS/Tw (TBS containing 0.05% v/v Tween 20 (8.17072.1000, Merck)). Samples, standards, and quality controls were diluted in TBS/Tw containing 5 mM EDTA and 0.1% w/v BSA, and subsequently added in duplicates. The plate was incubated at 37°C for 1 h. Wells were washed three times in TBS/Tw and incubated with biotinylated goat-*anti*-mouse Ig (1010-08, Southern Biotech), 1 μg/mL TBS/Tw, at 37°C for 1 h. Wells were washed 3 times in TBS/Tw, and Eu^3+^-labeled streptavidin (1244-360, PerkinElmer) diluted 1:1,000 in TBS/Tw containing 25 μM EDTA was subsequently added to the wells and incubated at RT for 1 h. Finally, the wells were washed three times with TBS/Tw, and 200 μL enhancement buffer (AMPQ99800, Amplicon) was added. The plate was shaken for 5 min and counts were read in a time-resolved fluorometry plate reader (Victor X5, PerkinElmer).

### Quantification and statistical analysis

GraphPad Prism v. 9 was used for statistical analyses. The nature of tests, n and meaning of bars, lines and error bars are indicated in the figure legends. Statistical significance is given as ∗*p* < 0.05; ∗∗*p* < 0.01; ∗∗∗*p* < 0.001; and ∗∗∗∗*p* < 0.0001, or ns, *p* > 0.05.
