# Supplementary material for: T-follicular regulatory cells expand to control germinal center plasma cell output but fail to curb autoreactivity
Source: iScience. 2024 Sep 4;27(10):110887. doi: 10.1016/j.isci.2024.110887 (PMC11417334; doi:10.1016/j.isci.2024.110887)
Supplement: Document S1. Figures S1–S9 [file mmc1.pdf]

iScience, Volume *27*

Supplemental information

T-follicular regulatory cells expand

to control germinal center plasma cell output

but fail to curb autoreactivity

Cecilia Fahlquist-Hagert, Thomas Rea Wittenborn, Mattias Krogh Pedersen, Lisbeth Jensen, and Søren Egedal Degn

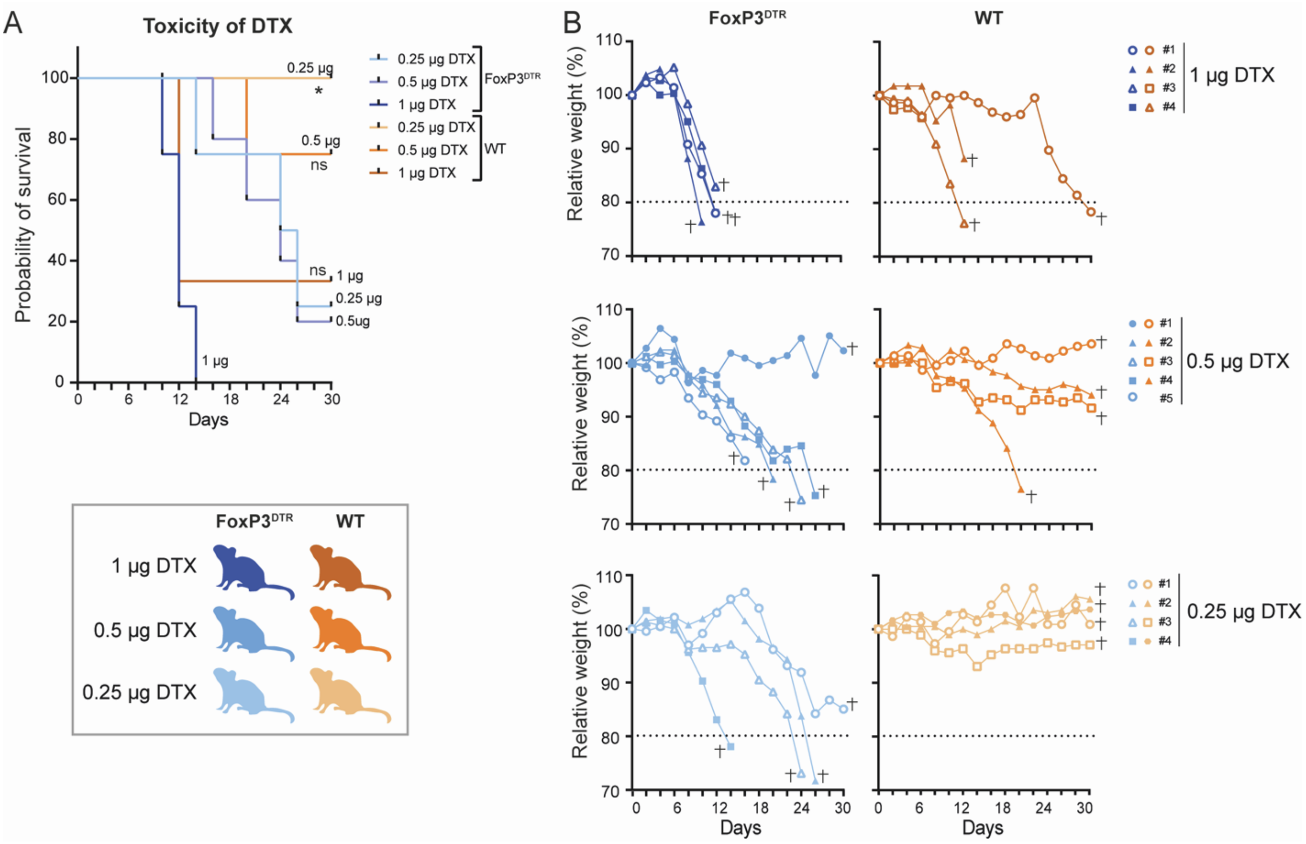

Figure S1. Dose titration of diphtheria toxin (DTX) in wild-type (WT) C57BL/6J mice and FoxP3-GFP/DTR (FoxP3^{DTR}) mice susceptible to depletion of FoxP3 cells, related to Figure 1. (**A**) Kaplan-Meyer plot for treatment of mice with 0.25 µg, 0.5 µg, or 1 µg DTX. The effect of the treatment is shown in probability of survival over the course of a month. Statistical comparison between survival curves comparing the genotypes/doses using Mantel Cox log-rank and Gehan-Breslow-Wilcoxon test. (**B**) Weight curves over the course of the treatment for individual mice in the groups relative to their starting weight (n=4-5).

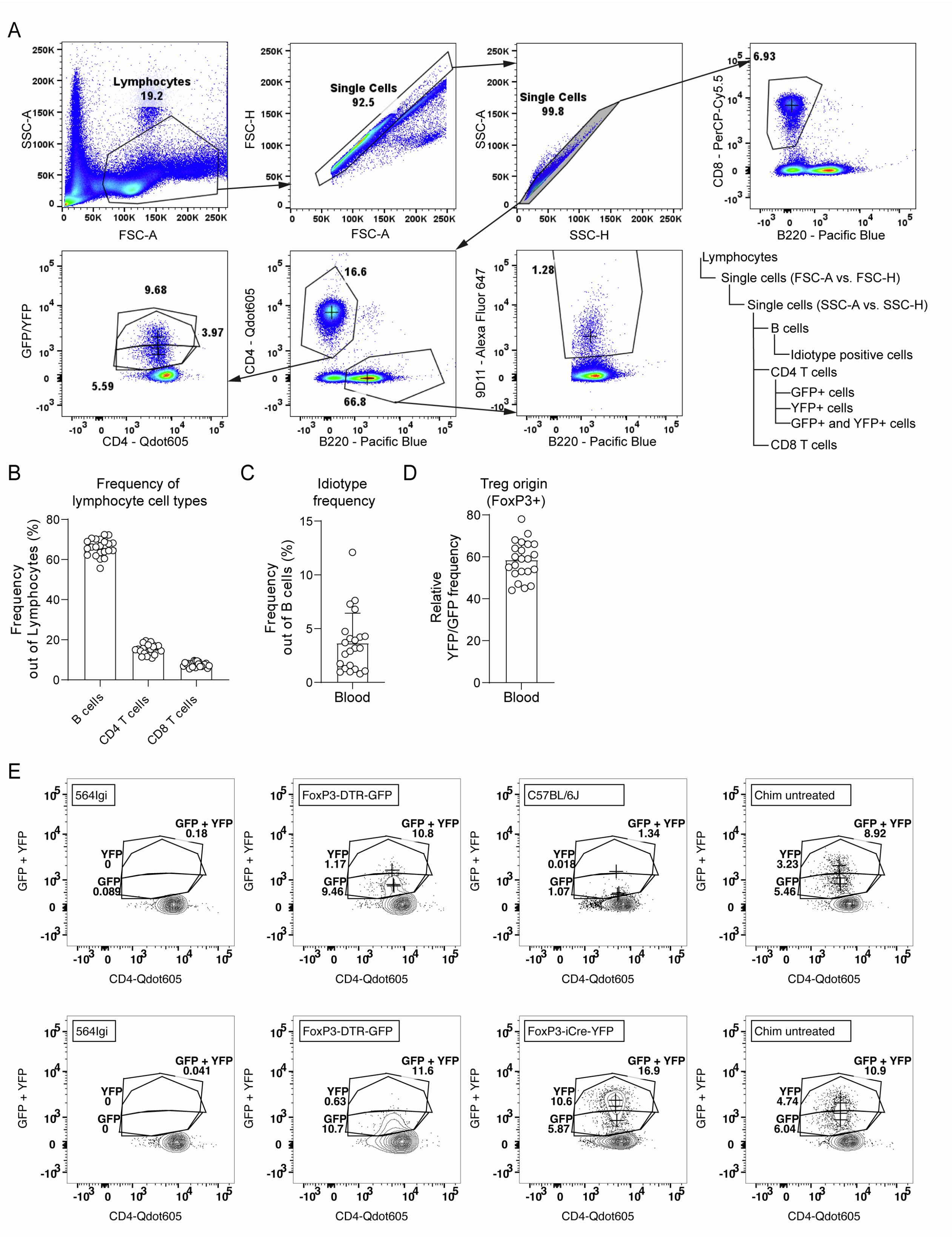

Figure S2. Blood immunophenotyping from mice presented in Figure 1 at six weeks post reconstitution. (**A**) Representative gating strategy. (**B**) frequencies of B, CD4 T and CD8 T cells. (**C**) Frequency of idiotype (9D11) positive B cells. (**D**) Relative ratio of YFP (Tfr block) over GFP (DTX-

ablatable) cells, reflecting the reconstitution with 564Igi-FoxP3-DTR/GFP versus FoxP3-iCre/YFP Bcl6$^{flx/flx}$ donor marrow. In B-C, n=22. (E) Examples of GFP versus YFP gating, including two 564Igi controls, two FoxP3-DTR-GFP controls, a C57BL/6J and a FoxP3-iCre-YFP control, and two untreated chimeras.

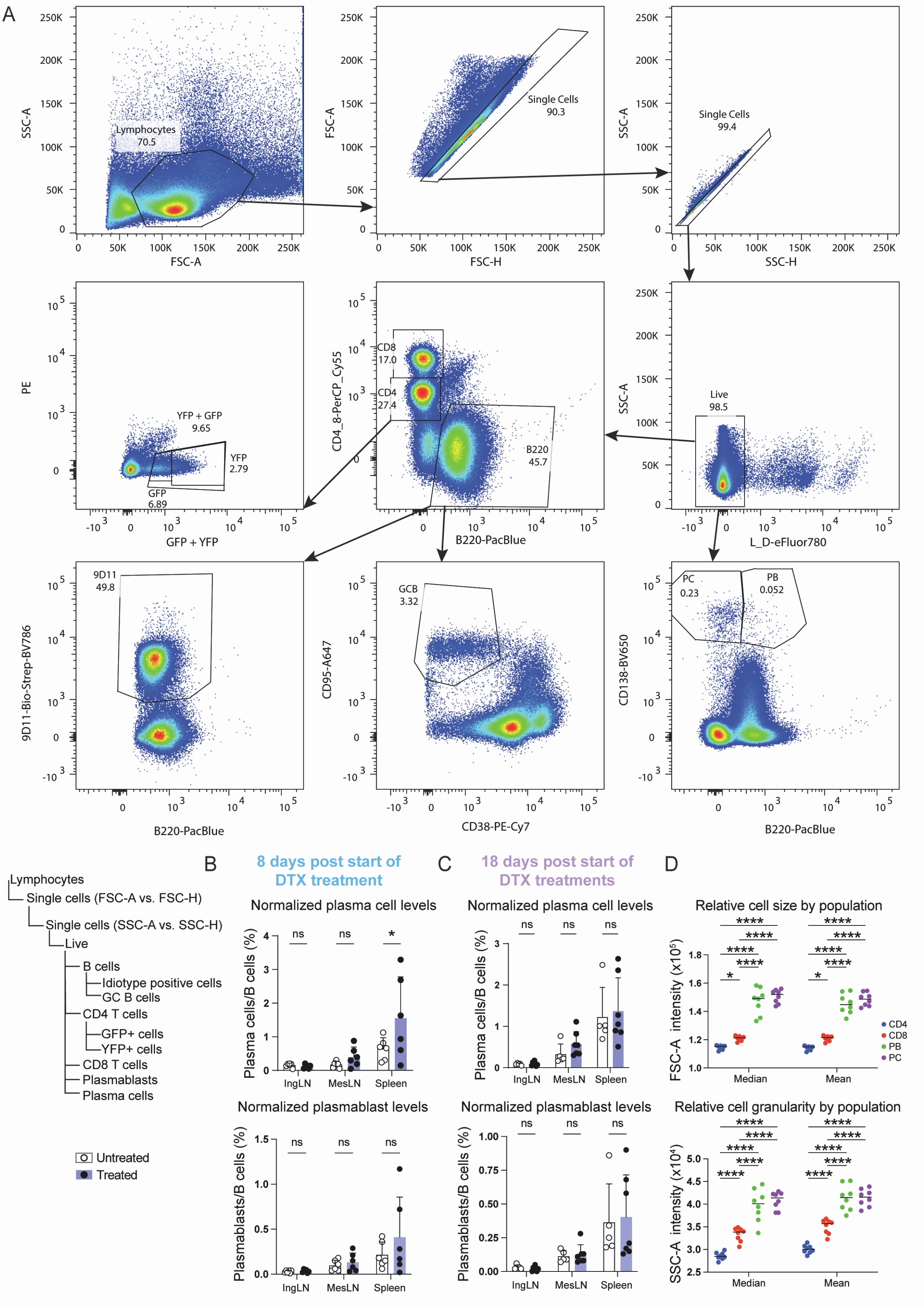

A
B
8 days post start of DTX treatment
C
18 days post start of DTX treatments
D
Lymphocytes 70.5
Single Cells 90.3
Single Cells 99.4
Live 98.5
CD8 17.0
CD4 27.4
B220 45.7
YFP + GFP 9.65
YFP 2.79
GFP 6.89
9D11 49.8
GCB 3.32
PC 0.23
PB 0.052
SSC-A
FSC-A
FSC-H
SSC-H
PE
GFP + YFP
CD4_8-PerCP_Cy55
B220-PacBlue
L_D-eFluor780
9D11-Bio-Strep-BV786
CD95-A647
CD38-PE-Cy7
CD138-BV650
Lymphocytes
Single cells (FSC-A vs. FSC-H)
Single cells (SSC-A vs. SSC-H)
Live
B cells
Idiotype positive cells
GC B cells
CD4 T cells
GFP+ cells
YFP+ cells
CD8 T cells
Plasmablasts
Plasma cells
Untreated
Treated
Normalized plasma cell levels
Plasma cells/B cells (%)
Normalized plasmablast levels
Plasmablasts/B cells (%)
IngLN
MesLN
Spleen
ns
*
Relative cell size by population
FSC-A intensity (x10^5)
Relative cell granularity by population
SSC-A intensity (x10^4)
Median
Mean
CD4
CD8
PB
PC
****

Figure S3. Supporting data for Figure 1. (**A**) Representative gating strategy for flow data presented in main Figure 1. Plasma cell (top row) and plasmablast (bottom row) levels from Figure 1 normalized to B cells at (**B**) 8 and (**C**) 18 days after start of DTX treatments. In B and C, bars represent mean + SD, and individual values are shown. (**D**) Individual values and mean for FSC-A (top) and SSC-A (bottom) intensity levels for CD4, CD8, PB, and PC populations as gated in panel A. Statistical significance given for two-way ANOVA with Šidák's post-test for B and C, and Holm- Šidák's post-test in D, ns (not significant) $p>0.05$; *$p<0.05$, ****$p<0.0001$.

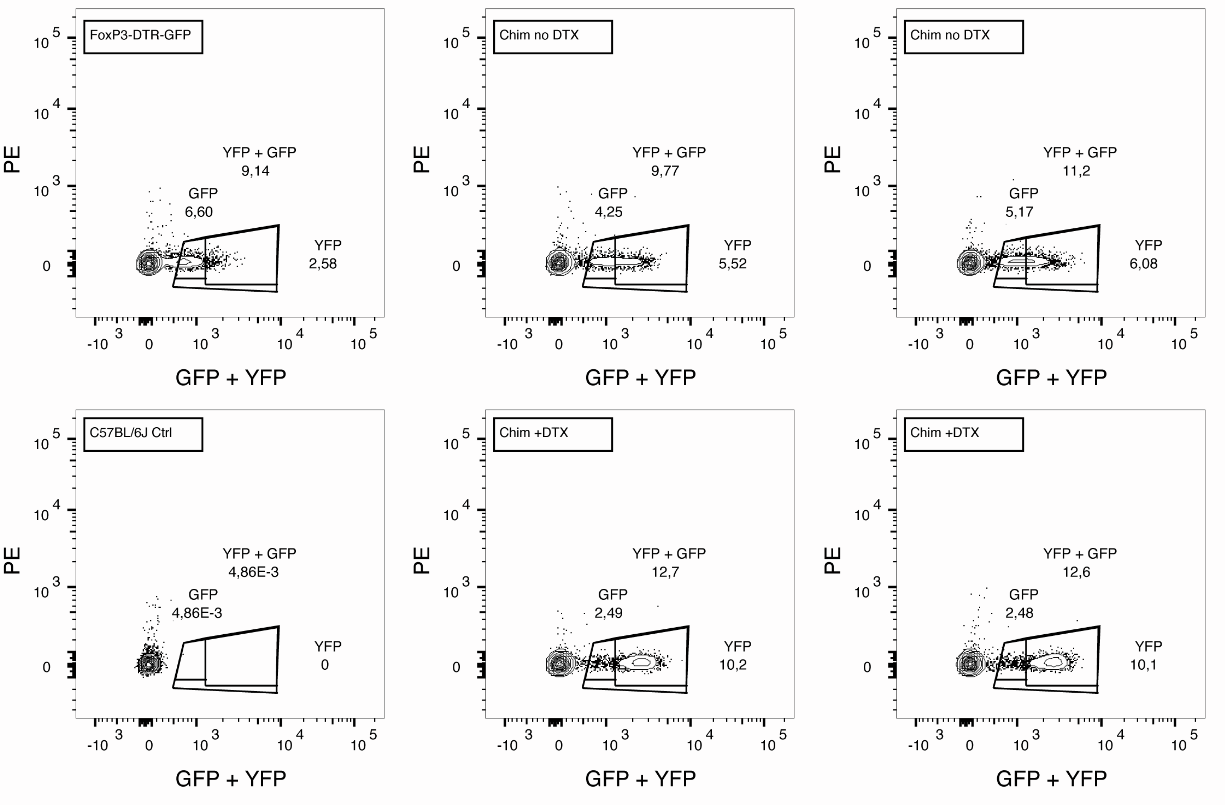

Figure S4. Examples of GFP versus YFP gating for tissue analyses presented in Figure 1. Includes a FoxP3-DTR-GFP control, a C57BL/6J control, two untreated, and two DTX treated chimeras.

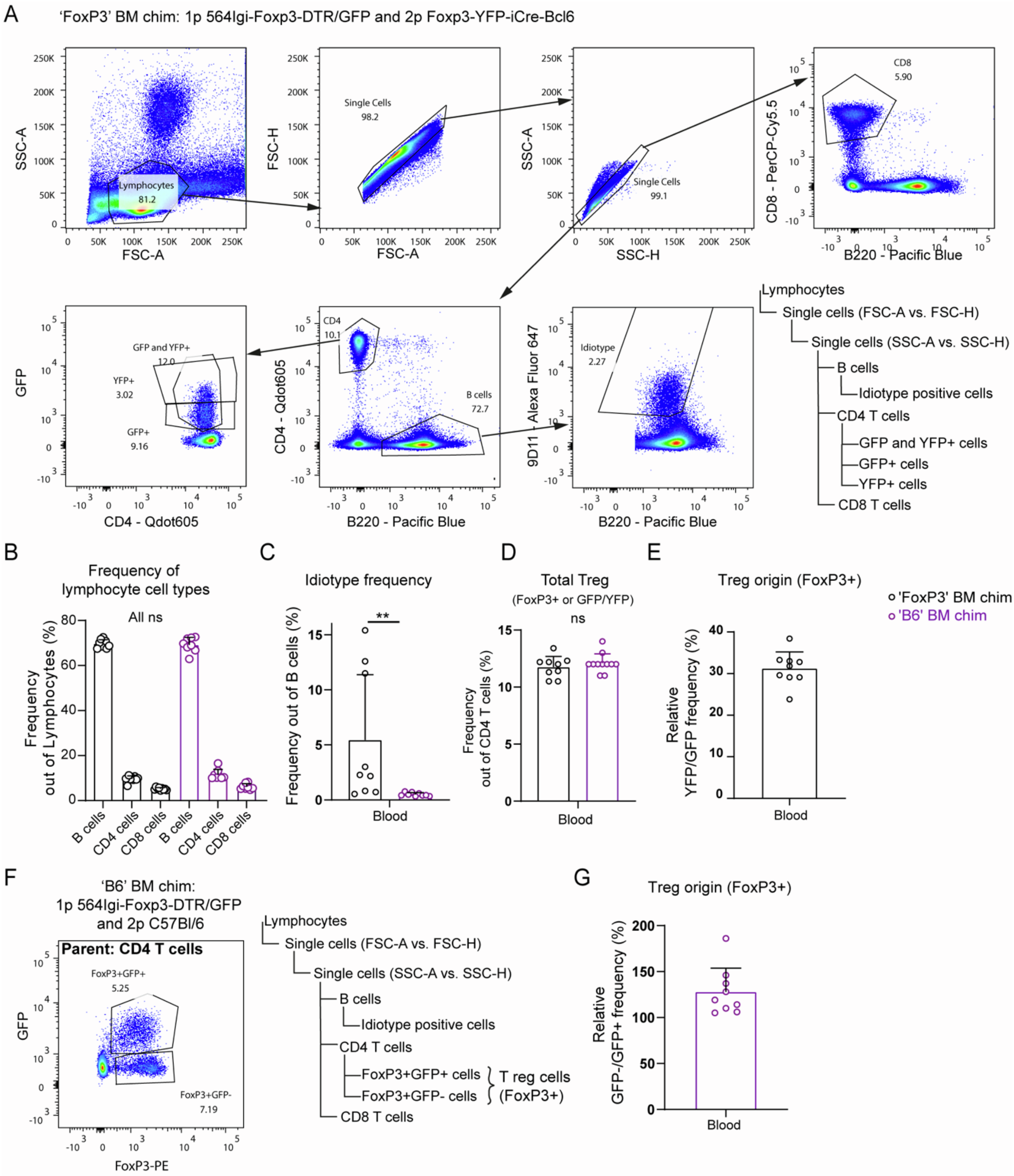

Figure S5. Immunophenotyping of blood from mice presented in Figure 2 at six weeks post reconstitution. (**A**) Representative gating strategy. (**B**) frequencies of B, CD4 T and CD8 T cells. (**C**) Frequency of idiotype (9D11) positive B cells. (**D**) Frequency of DTX-ablatable (GFP+) cells. (**E**) Treg origin in FoxP3 chimeras expressed as percentual fraction of YFP+ cells over GFP+ cells. (**F**) gating of FoxP3+GFP+ vs. FoxP3+GFP- cells in B6 chimeras. (**G**) Treg origin in B6 chimeras expressed as percentual fraction of GFP- over GFP+ cells. In B-E, n=9 for FoxP3 and n=11 for B6 chim. In G, n=9 for B6 chim. In B-E and G, bar graphs represent mean + SD, and individual values are shown.

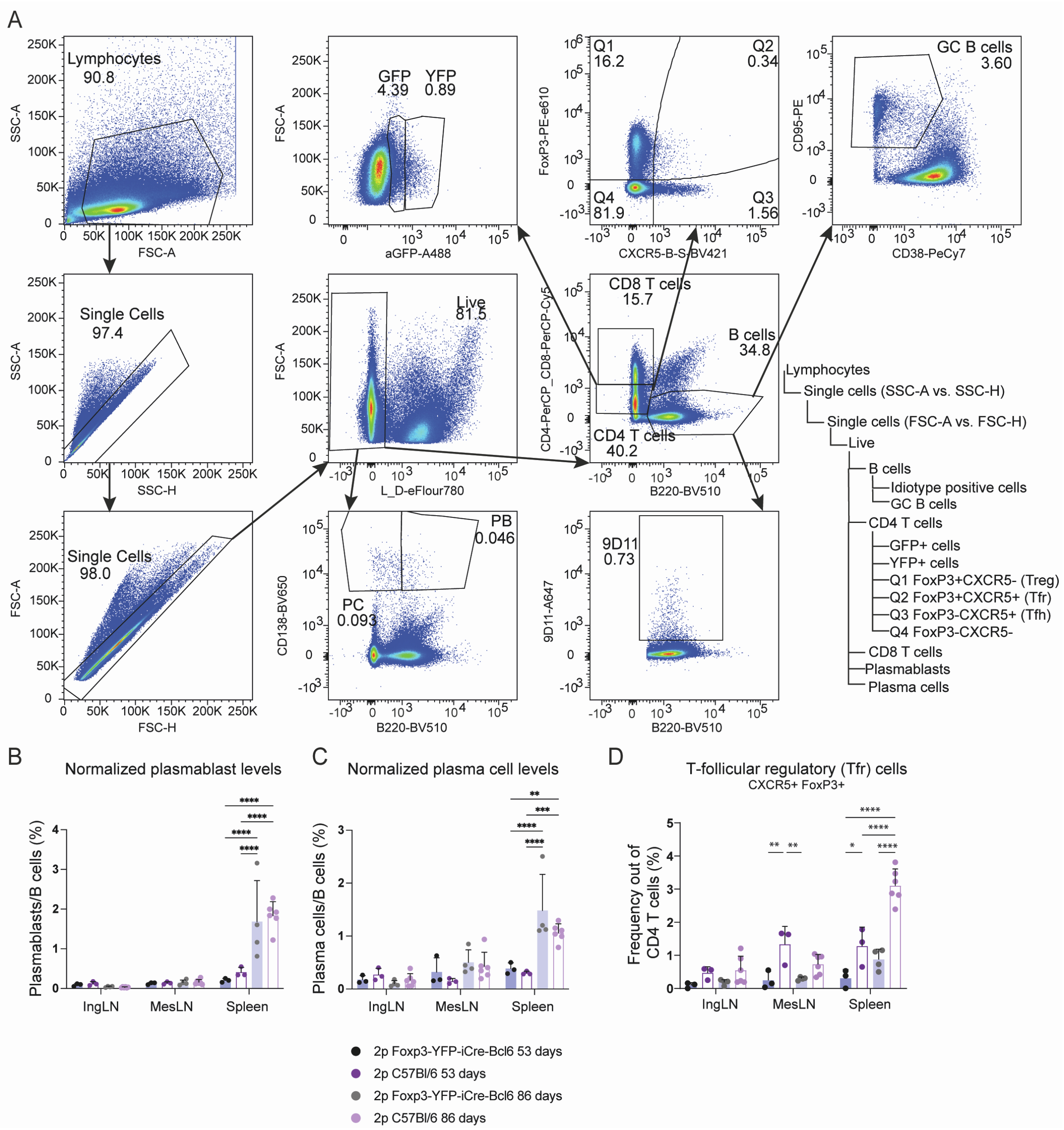

Figure S6. Supporting data for Figure 2. (**A**) Representative gating strategy for flow data presented in main Figure 2. (**B**) Plasmablast levels from Figure 2 normalized to B cells. (**C**) Plasma cell levels from Figure 2 normalized to B cells. (**D**) Frequency of T-follicular regulatory cells (CXCR5+FoxP3+ out of CD4 T cells). In B-D bar graphs represent mean + SD, and individual values are shown. Statistical significance given for two-way ANOVA with Tukey's posttest in B-C. Only significant comparisons are shown: *p<0.05; **p<0.01; ***p<0.001; ****p<0.0001, all other comparisons were not significant (ns, p>0.05).

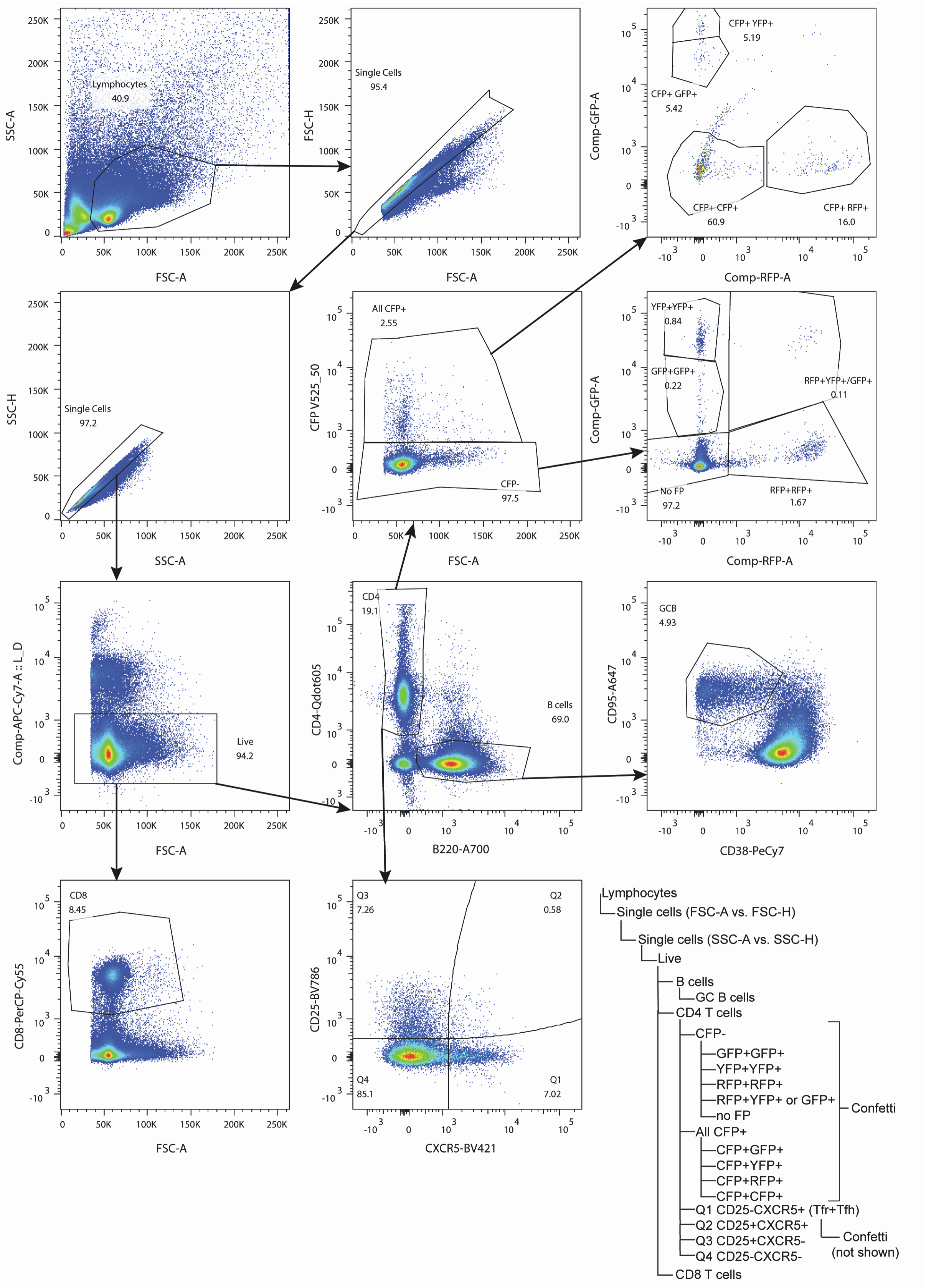

Figure S7. Gating strategy for data presented in Figure 3. Confetti subgating for CD25-CXCR5+ (Tfh+Tfr) subset of CD4 T cells is not shown for space constraints, but is identical to that presented for CD4 T cells. Of note, activation of the Confetti color cassette is FoxP3 dependent.

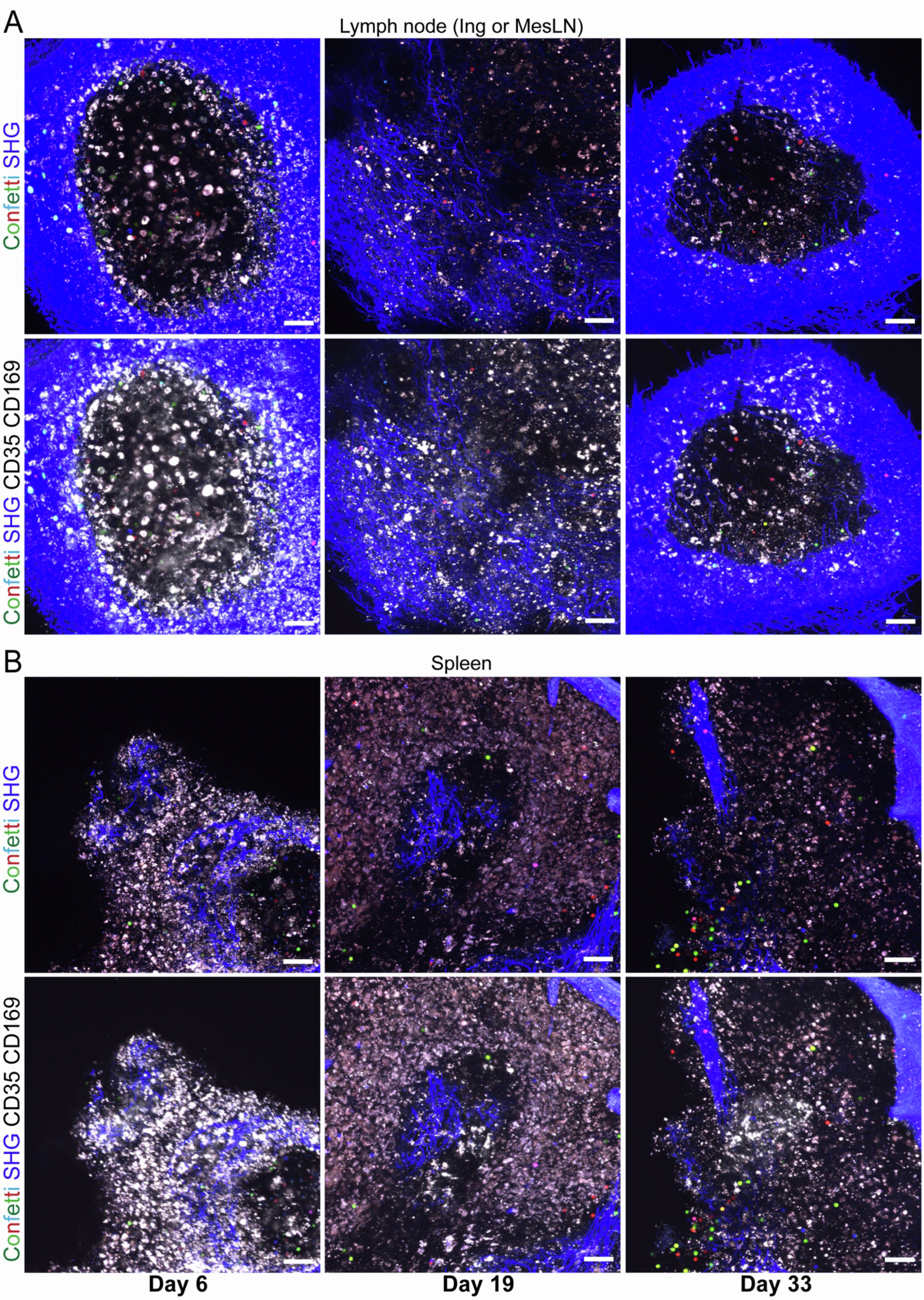

Figure S8. Supplemental data for Figure 4, FoxP3 BM chimera group. Micrographs representing ex vivo lymph node (**A**) and spleen (**B**) samples from day 6, 19 and 33. The micrograph shows second

harmonic generation (SHG, blue) and confetti cells only (top row) or combined with the signal from CD35 stain (bottom row, white) to indicate the germinal center. Scale bars in A and B represent 50 μm.

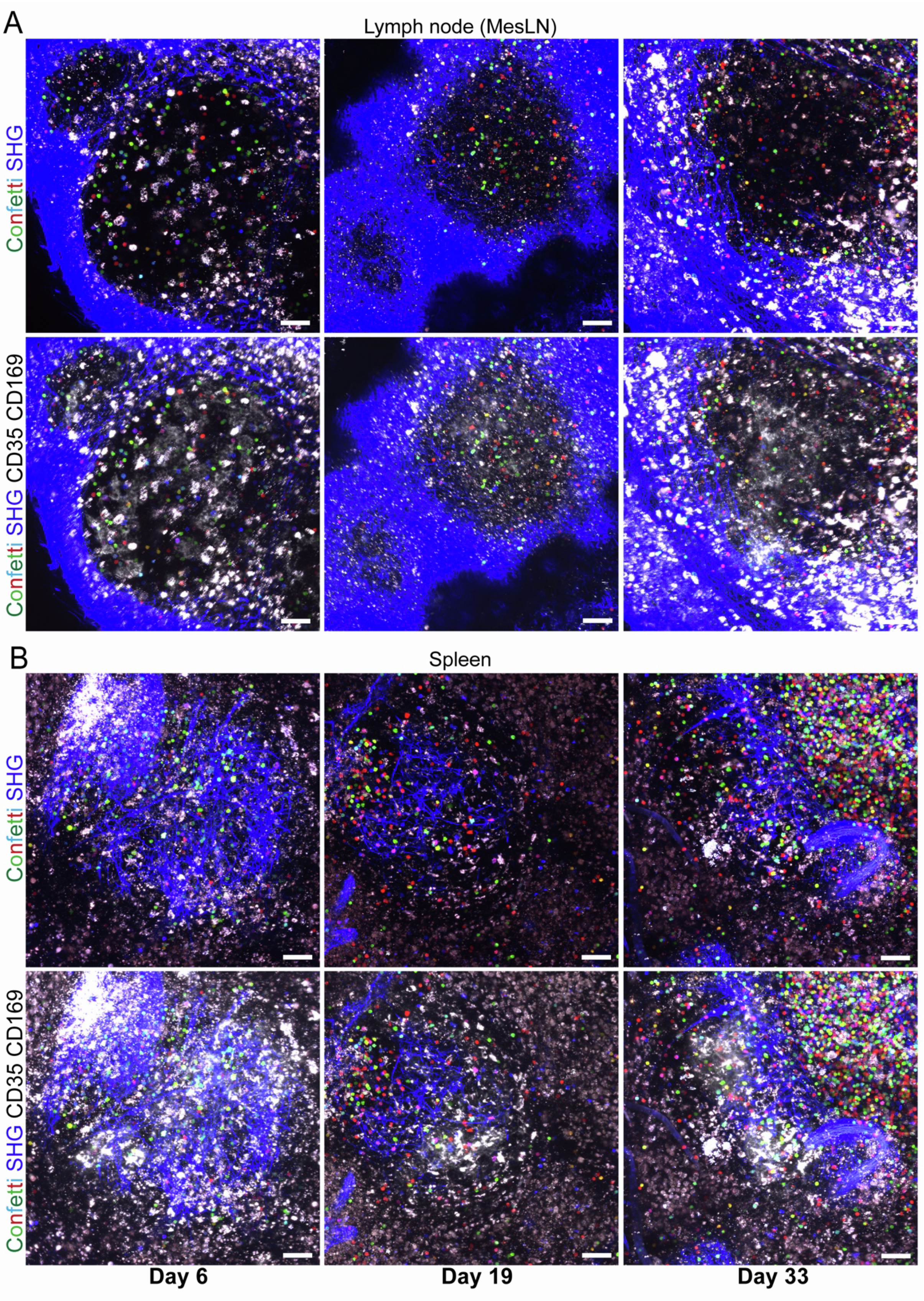

Figure S9. Supplemental data for Figure 4, CD4 BM chimera group. Micrographs representing ex vivo mesenteric lymph node (**A**) and spleen (**B**) samples from day 6, 19 and 33. The micrograph shows

second harmonic generation (SHG, blue) and confetti cells only (top row) or combined with the signal from CD35 stain (bottom row, white) to indicate the germinal center. Scale bars in A and B represent 50 μm.
